# Nuclear GSK-3β and Oncogenic KRas Lead to the Retention of Pancreatic Ductal Progenitor Cells Phenotypically Similar to Those Seen in IPMN

**DOI:** 10.3389/fcell.2022.853003

**Published:** 2022-05-13

**Authors:** Li Ding, Kaely Roeck, Cheng Zhang, Brooke Zidek, Esther Rodman, Yasmin Hernandez-Barco, Jin-San Zhang, William Bamlet, Ann Oberg, Lizhi Zhang, Nabeel Bardeesy, Hu Li, Daniel Billadeau

**Affiliations:** ^1^ Division of Oncology Research, College of Medicine, Mayo Clinic, Rochester, MN, United States; ^2^ Department of Molecular and Experimental Therapeutics, College of Medicine, Mayo Clinic, Rochester, MN, United States; ^3^ Center for Cancer Research, Harvard Medical School, Boston, MA, United States; ^4^ Center for Precision Medicine, The First Affiliated Hospital of Wenzhou Medical University, Wenzhou, China; ^5^ Department of Health Sciences Research, College of Medicine, Mayo Clinic, Rochester, MN, United States; ^6^ Department of Laboratory Medicine and Pathology, College of Medicine, Mayo Clinic, Rochester, MN, United States

**Keywords:** GSK-3β, KRAS, AQP5, progenitor cell, intraductal papillary mucinous neoplasm, pancreatic adenocarcinoma

## Abstract

Glycogen synthase kinase-3β (GSK-3β) is a downstream target of oncogenic KRas and can accumulate in the nucleus in pancreatic ductal adenocarcinoma (PDA). To determine the interplay between oncogenic KRas and nuclear GSK-3β in PDA development, we generated Lox-STOP-Lox (LSL) nuclear-targeted GSK-3β animals and crossed them with LSL-KRas^G12D^ mice under the control of the Pdx1-cre transgene—referred to as KNGC. Interestingly, 4-week-old KNGC animals show a profound loss of acinar cells, the expansion of ductal cells, and the rapid development of cystic-like lesions reminiscent of intraductal papillary mucinous neoplasm (IPMN). RNA-sequencing identified the expression of several ductal cell lineage genes including AQP5. Significantly, the Aqp5^+^ ductal cell pool was proliferative, phenotypically distinct from quiescent pancreatic ductal cells, and deletion of AQP5 limited expansion of the ductal pool. Aqp5 is also highly expressed in human IPMN along with GSK-3β highlighting the putative role of Aqp5^+^ ductal cells in human preneoplastic lesion development. Altogether, these data identify nGSK-3β and KRas^G12D^ as an important signaling node promoting the retention of pancreatic ductal progenitor cells, which could be used to further characterize pancreatic ductal development as well as lineage biomarkers related to IPMN and PDA.

## Introduction

Pancreatic ductal adenocarcinoma (PDA) is predicted to be the second leading cause of cancer-related deaths in the United States by 2030. The 5-year relative survival rate of all stages combined is less than 10%, with 80% of patients having a locally advanced tumor at diagnosis, which highlights the significant need for the development of early-detection strategies and models to understand PDA tumor initiation and development (Rahib et al., 2014; Garrido-Laguna and Hidalgo, 2015; American Cancer Society, 2019; Blackford et al., 2020). Genetically engineered mouse models of PDA have served an important role in defining the mechanisms contributing to PDA initiation, progression, and metastasis (Bardeesy and DePinho, 2002; Reichert and Rustgi, 2011; Storz and Crawford, 2020). Oncogenic mutations in KRas represent the earliest and most prevalent mutation thereby highlighting its role as a driver of PDA (Morris et al., 2010; Kanda et al., 2012). Importantly, pancreas-specific, cre-mediated activation of a knockin allele of the most common mutation, KRas^G12D^, leads to the development of early preneoplastic lesions and late-onset PDA, which can be accelerated by loss of tumor suppressors such as TP53 or p16 or experimentally by inducing pancreatitis (Hingorani et al., 2003; Guerra et al., 2007; Reichert and Rustgi, 2011).

Glycogen synthase kinases, GSK-3α and GSK-3β, were originally identified as key enzymes regulating glycogen metabolism (Woodgett, 1990; Kaidanovich-Beilin and Woodgett, 2011; Cormier and Woodgett, 2017). However, accumulating evidence has suggested a role for these kinases in several human malignancies (McCubrey et al., 2014; Beurel et al., 2015; Walz et al., 2017) and has identified them as therapeutic targets in pancreatic cancer (Ding and Billadeau, 2020). Significantly, GSK-3β is overexpressed in PDA and aberrant nuclear accumulation, as well as increased mRNA expression of GSK-3β, has been associated with high-grade tumors (Ougolkov et al., 2006; Xie et al., 2017). Moreover, our prior studies showed that mutant KRas increases GSK-3β gene expression *in vivo* and *in vitro* (Zhang et al., 2011; Ding et al., 2017). However, the studies conducted so far to address the multifaceted role of GSK-3β in pancreatic cancer *in vivo* have mostly relied on loss-of-function approaches including knockout mice or GSK-3 inhibitors (Baumgart et al., 2016; Ding et al., 2017; Ding and Billadeau, 2020). The role of nuclear GSK-3β *in vivo*, particularly as it pertains to PDA precursor lesion development, is unknown.

To address this, we generated a mouse harboring an inducible nuclear-targeted GSK-3β transgene, which we crossed with KC (KRas^G12D^; Pdx1-Cre) mice to activate the transgenes during early pancreatic development (referred to as KNGC). Unexpectedly, we observed profound pancreatic cyst development and expansion of cytokeratin-19^+^ ductal cells in 4-week-old KNGC mice that accumulated with age. Bulk RNA-sequencing data indicated that nuclear GSK-3β and KRas^G12D^ reprogrammed pancreatic progenitor cells toward the ductal lineage, resulting in a loss of acinar cells and the expansion of ductal cells with expression of Aquaporin 5 (AQP5). Single-cell RNA-sequencing (scRNA-seq) indicated that the ductal cell cluster not only expressed AQP5 but also several other genes recently identified in human and murine pancreatic ductal precursors. Our data suggest that nuclear GSK-3β and KRas^G12D^ cooperate to promote the retention of a ductal progenitor cell pool in the pancreas which could be used to further define this progenitor cell population and its contribution to preneoplastic lesion development.

## Materials and Methods

### Generation of Nuclear GSK-3β Conditional Knock-In Mice and Mouse Lines

Conditional GSK-3β knock-in mice containing an SV40 nuclear localization sequence (NLS; nGSK-3β) and HA-tag were generated by the Transgenic and Gene Knockout Core at the Mayo Clinic according to established protocols (Dawlaty and van Deursen, 2006). The nGSK-3β targeting construct was generated using the previously described Rosa26-targeting vector pR26-CAG/EGFP-Asc, which contains a CAG promoter, and loxP flanked Neo/Stop cassette. LSL-KRas^G12D^, Pdx1-Cre and GSK-3β^F/F^ mice have previously been described (Hingorani et al., 2003; Ding et al., 2017). Pancreas specific expression of nuclear GSK-3β with KRas^G12D^ was generated by crossing Pdx1-cre/LSL-KRas^G12D^ (KC) mice with LSL-nuclear GSK-3β (NG) mice to produce Pdx1-cre/LSL-KRas^G12D^/LSL-nuclear GSK-3β (KNGC) animals. Additional Ptf1a/p48-cre mice was obtained from Dr. Martin Fernandez-Zapico (Mayo Clinic, Rochester, MN, United States). Aqp5 knockout mice were obtained from Dr. Varadaraj Kulandaiappan (Stony Brook University, NY, United States). All experiments were performed with littermate-matched pairs, both male and female, and were approved by the Mayo Clinic Institutional Animal Care and Use Committee.

### Pancreatic Ductal Cell Isolation

Preparation of single-cell suspensions from the mouse pancreas and pancreatic ductal cell isolation was performed as described (Li et al., 2013; Reichert et al., 2013; Elyada et al., 2019). Cells were gently pipetted to maximize the release of single cells and spun down and resuspended in ACK lysis buffer to eliminate red blood cells. ACK buffer was quenched with 2% fetal bovine serum (FBS) in PBS. Immune and stroma cell depletion was performed by EasySep™ Mouse Streptavidin RapidSpheres™ Isolation Kit (STEMCELL Technologies Inc., Cambridge, MA, United States) with Biotin-labeled anti-mouse CD45.1 and anti-podoplanin antibodies. Negatively-selected cells were resuspended in a sorting buffer containing PBS, 0.5% bovine serum albumin, and 2 mM EDTA, followed by 10 min incubation with fluorescein-labeled Dolichos biflorus agglutinin (DBA) (Vector Laboratories) with agitation at 4°C. Cells were washed in the sorting buffer and resuspended in the same buffer with anti-FITC Microbeads (Miltenyi Biotec), and incubated on a rotor for 15 min at 4°C. Separation was performed using MS columns (Miltenyi Biotec), according to the manufacturer’s protocol.

### Immunoblot Analysis

Collected pancreata or isolated pancreatic ductal cells were lysed with Western lysis buffer (1% Triton X-100, 10 mM Tris Base, 50 mM NaCl, 5 mM EDTA, 50 mM NaF, 30 mM Na4P2O7 pH 7.4) supplemented with aprotinin, leupeptin, sodium orthovanadate, phenylmethylsulfonyl fluoride (PMSF) and calyculin A (Cell Signaling Technologies, Beverly, MA, United States). Antibodies used for immunoblotting and immunofluorescence are described in detail in Supplementary Table S1.

### IHC, EdU Labeling and Immunofluorescence

Mice were anesthetized using isoflurane (Nova Plus Pharmaceuticals), followed by cervical dislocation. The whole pancreas was quickly removed and fixed overnight in 4% PFA with gentle shaking, embedded in paraffin, cut into 5 μm-thick sections. Sections were subjected to H&E, IHC and immunofluorescence staining as described (Ding et al., 2017). Anti-rabbit secondary (#8114, Cell Signaling Technologies, Beverly, MA, United States) and diaminobenzidine substrate kit (#8059, Cell Signaling Technologies, Beverly, MA, United States) were used for immunohistochemistry. Slides were then counterstained with Mayer’s Hematoxylin before dehydration and mounting. For immunofluorescence, slides were incubated with fluorescent-conjugated secondary antibodies for 1 h at room temperature before confocal scanning. For EdU labeling, mice were injected with EdU at a concentration of 50 mg/kg/bw in saline 2 h before sacrifice. Staining was performed by Click-iT^®^ EdU Alexa Fluor^®^ 647 Imaging Kit following manufacturer’s instructions (Thermofisher scientific, United States). Confocal images were collected with an LSM-800 laser scanning confocal microscope with a 63×-oil Plan-Apochromat objective lens using ZEN Blue 2.6 software package (Carl Zeiss, Oberkochen, Germany). For whole pancreas tissue section scanning, stage marks were placed around the edge of the pancreas and tile region was drawn to cover all the stage marks. Images were taken under ×10 objective lens using tiles. The stitching method within the Zen Blue software package was applied to process the tile images into one final image. The percentage of EdU-647 + cells was enumerated, and the area and integrated density of indicated staining were measured using the ImageJ open-source image-processing package.

### RNA Isolation, Quantitative RT-PCR and RNA-Seq Data Analysis

RNA isolation and quantitative RT-PCR were performed as previously described (Ding et al., 2017). Detailed information on preparation of the RNA and cells for RNA-seq and single-cell RNA-seq and data analysis can be found in Supplementary Material and Methods.

### Staining of Tissue Microarrays

Tissue microarrays (TMAs) were acquired from the Mayo Clinic SPORE in Pancreatic Cancer and consists of 140 unique individuals with IPMN. IHC for Aqp5 and GSK-3β, and immunofluorescence staining of Agr2/DBA/CK19 were performed as described above. Cores were excluded if absent in the slide. Aqp5 and GSK-3β histological scoring was performed and evaluated by a pathologist as follows, staining intensities were scored from 0 (no staining) to 3 (high staining) and extent was scored from 0 (negative), 1 (<25%), 2 (25%–50%), 3 (50%–75%), to 4 (>75%, widespread staining). Whole slides scanning and measurement of intensity and area of Agr2/DBA/CK19 immunofluorescence staining in TMAs were described above and staining intensities were scored from weak (<33%), medium (33%–66%) to strong (>66%) and extent was scored as the percentage of Agr2/DBA in CK19 positive cells. The H-score system was used for IHC evaluation of Aqp5, GSK-3β and IF evaluation of Agr2 and DBA by multiplying the extent or percentage of cells with staining intensity ordinal value ranging from 0 to 300. H-scores were available on 133–135 of the 140 patients represented on the TMA.

### Statistical Analysis

Data are expressed as mean ± SEM and analyzed by repeated measures analysis of variance, one-way ANOVA and unpaired Student’s *t*-test using GraphPad Prism software (GraphPad Software Inc., La Jolla, CA, United States). A value of *p* < 0.05 denotes statistical significance. Scatterplots including a loess smoother (smoothing parameter = 0.8) were generated to visualize the relationship between Aqp5, GSK-3β, Agr2 and DBA-FITC. Spearman correlation coefficient and *p*-value are reported to summarize the nature of the relationship.

## Results

### GSK-3β Ablation Limits KRas^G12D^-Induced Pancreatic Cancer Development

We have previously demonstrated that GSK-3β gene expression is a target of oncogenic KRas signaling in pancreatic cancer cell lines and pancreas-specific deletion of GSK-3β in KRas^G12D^ mice reduces cearulein-induced ADM and PanIN lesion development (Zhang et al., 2011; Ding et al., 2017). To examine the impact of GSK-3β ablation on pancreatic cancer progression, the pancreas was collected from 8- to 10-month-old Pdx1-cre (Wildtype, WT), Pdx1-cre/GSK-3β^F/F^ (KO), Pdx1-cre/LSL-KRas^G12D^ (KC) and Pdx1-cre/LSL-KRas^G12D^/GSK-3β^F/F^ (RKO) littermates (Supplementary Figure S1A, upper panel). Consistent with our previous observation (Ding et al., 2017), there were no significant differences between WT and KO mice at these ages (Supplementary Figure S1A, lower panels). The total number of ductal lesions and their grade were scored in representative pancreatic sections. Depletion of GSK-3β was confirmed by immunofluorescent staining of GSK-3β and the pancreatic ductal cell marker CK19. Strong GSK-3β signal was detected in CK19^+^ neoplastic ducts from KC mice but not RKO mice (Supplementary Figure S1B). Consistent with a previous report (Hingorani et al., 2003), most pancreas tissue from 9-month-old KC mice were replaced by metaplastic ducts, which were surrounded by desmoplasia (Supplementary Figure S1C). In contrast, RKO mice had diminished numbers of ADM and low-grade PanIN lesions (PanIN-1 and -2), and reduced incidence of high-grade PanIN-3 and invasive cancer (Supplementary Figures S1C,D). We next examined the proliferation of neoplastic ducts in KC and RKO mice. As shown in Supplementary Figures S1E,F, we observed an accumulation of EdU^+^/CK19^+^ ductal cells within neoplastic areas in KC mice, which were dramatically reduced in RKO mice. These data suggest that GSK-3β is required for KRas^G12D^-driven cell proliferation and deletion of GSK-3β impairs preneoplastic lesion and pancreatic cancer development in KC mice.

### Nuclear GSK-3β and KRas^G12D^ Promote Pancreatic Ductal Cell Expansion and IPMN Development

**FIGURE 1:**
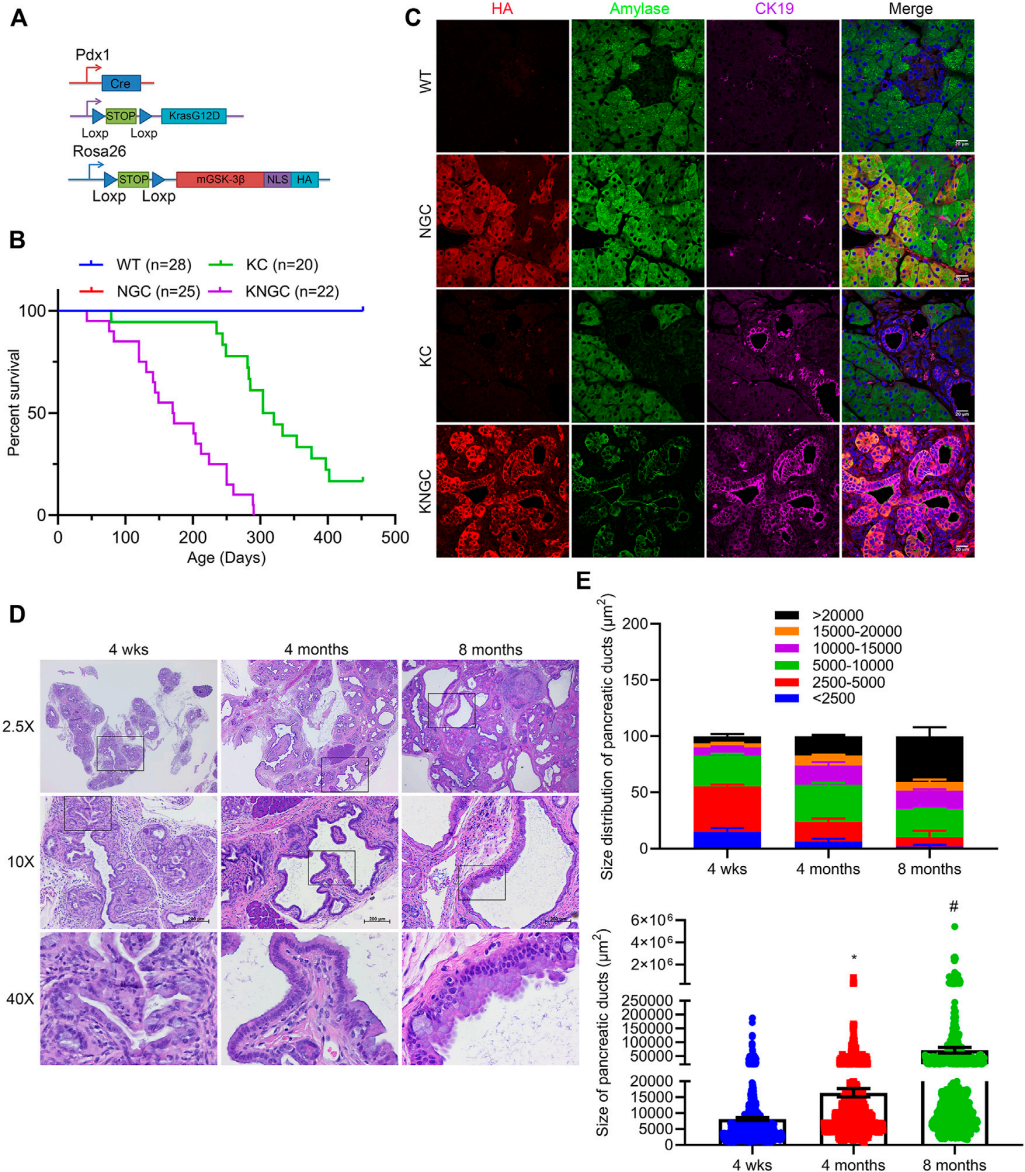
Nuclear GSK-3β and KRas^G12D^ promote pancreatic ductal cell expansion and IPMN development. **(A)** Schematic representation of KNGC (LSL-KRas^G12D^/Rosa26-LSL-nuclear GSK-3β/Pdx1-Cre) mouse model. Blue triangles indicate loxP sites. **(B)** Kaplan-Meier survival curve of the indicated genotypes. **(C)** Immunofluorescence (IF) staining of HA (red), amylase (green) and CK19 (purple) from pancreatic sections of indicated genotypes at 4 weeks age. Nuclei were counter-stained with Hoechst (blue). Shown are representative image from three different mice. **(D)** H&E-stained pancreatic sections from KNGC mice of 4-week, 4-month, and 8-month. Black boxes indicate magnified area. Bars = 200 μm. **(E)** Quantification of pancreatic duct size distribution and distribution of pancreatic duct size for KNGC mice were analyzed and expressed as mean ± SEM. *n* = 5. ∗*p* < 0.05 4-month versus 4-week KNGC mice. ^#^ *p* < 0.05 8-month versus 4-month KNGC mice.

We have previously shown that GSK-3β is overexpressed in PDA and becomes localized to the nucleus in high-grade tumors (Ougolkov et al., 2006). To characterize the role of nuclear GSK-3β in pancreatic cancer development, we generated a pancreas-specific nuclear-targeted GSK-3β expression model by inserting a GSK-3β cDNA transgene containing an HA tag and a nuclear localization signal sequence into the *Rosa26* locus. The transgene also contained a 5′ Lox-STOP-Lox (LSL) cassette for tissue-specific activation. Mice carrying LSL-nuclear GSK-3β (NG) were crossbred with KC mice to produce KNGC mice (Figure 1A). As shown in Supplementary Figure S2A, protein extracts from both NGC and KNGC animals showed a unique HA band which was not seen in WT or KC mice. Overexpression of GSK-3β in NGC and KNGC mice was also validated by immunohistochemistry staining (Supplementary Figure S2B). Phosphorylation of Erk1/2, a downstream target of activated KRas, was only slightly upregulated in KC mice due to limited formation of ADM and PanIN precursor lesions at this age (Hingorani et al., 2003). However, KNGC mice had a dramatic increase of phospho-Erk1/2 (Supplementary Figure S2A), suggesting a hyper-activation of KRas signaling induced by expression of nuclear GSK-3β. Consistent with the immunoblot results, gross pathological examination and H&E staining revealed hallmarks of pancreatic neoplastic transformation, including visible cysts and desmoplasia in 4-week-old KNGC mice, but no gross pathological changes nor abnormal pancreas architecture were found in the pancreas from 4-week-old WT (Cre-expressing), NGC or KC mice of similar age (Supplementary Figures S2C,D).

Immunofluorescent staining confirmed that the HA-tag was widely expressed in acinar and ductal cells within the pancreas of NGC and KNGC mice, which was absent from WT and KC mice (Figure 1C). Due to apparent loss of normal physiologic function of the pancreas, KNGC mice had a significantly lower body weight across their lifespan and a dramatically shortened median survival of approximately 6 months as compared with KC mice (Figure 1B; Supplementary Figures S2E,F). Histologically, normal acinar cell clusters were rarely seen in KNGC mice (Supplementary Figure S2C). At 4 weeks of age, KNGC mice developed multifocal ductal structures and atypical ductal cells across the pancreas, which expanded with age (Figures 1D,E). Loss of ductal cell polarity, papillary architecture with increased nuclear/cytoplasmic ratio, and mucinous epithelial neoplasm representing human intraductal papillary mucinous neoplasm (IPMN) as reviewed by a pathologist, were most frequently seen in 8-month-old KNGC mice, whereas no invasive cancer or metastases were observed in this age group of animals. It is of interest that NGC mice were indistinguishable from WT littermates at any age (data not shown), suggesting that nuclear GSK-3β expression, on its own, is insufficient to drive ductal cell expansion.

Given the dramatic phenotype in 4-week old KNGC mice, and the fact that expression of PDX-1/IPF1 is believed to be the earliest identifiable marker in pancreatic progenitor cells starting from E8.5-E9.5 (Song et al., 1999; Kim and MacDonald, 2002; Hingorani et al., 2003), we examined the pancreata of neonatal KNGC mice. Interestingly, we observed decreased acinar area, increased ductal area, ductal hyperplasia and enlargement (Supplementary Figures S3A,B). Ptf1a/p48 is another essential transcription factor for pancreas development that is expressed at early stages in the progenitors of pancreatic ducts, exocrine and endocrine cells from E10-E11.5, making Ptf1a/p48 another commonly used promoter for Cre-driver lines in pancreas research (Kawaguchi et al., 2002; Magnuson and Osipovich, 2013). It is interesting that some studies found preserved pancreas formation using p48-cre due to its limited Cre-expression within the early pancreas compared to PDX1^early^-cre (Heiser et al., 2008). Thus, we next generated KNGp48-cre mice using Ptf1a/p48-cre mice (Supplementary Figure S3C), and strikingly, we observed significant phenotype of ductal hyperplasia and papillary architecture in 4-week old KNGp48-cre mice to similarly aged KNGC (Supplementary Figure S3D). To further investigate the role of nuclear GSK-3β in driving the phenotype in KNGC mice, we crossed KNGC mice with GSK-3β^F/F^ mice to generate KNGC mice in an endogenous GSK-3β knock-out background (KNGCGko, Supplementary Figure S3E). Immunoblot results confirmed the expression of KRasG12D and HA-tag in KNGC and KNGCGko mice and loss of GSK-3β protein in KO, RKO and KNGCGko mice (Supplementary Figure S3F). Interestingly, GSK-3β knockout did not affect ERK phosphorylation or expansion of ductal cells in KNGC mice (Supplementary Figures S3F,G). Taken together, these data suggest that induced nuclear GSK-3β expression by either PDX1 or p48 promotes the expansion of a ductal cell compartment when combined with oncogenic KRasG12D.

### Transcriptional Regulation of Pancreatic Ductal Neoplasm by Overexpression of Nuclear GSK-3β and Oncogenic KRas^G12D^ Activation

**FIGURE 2:**
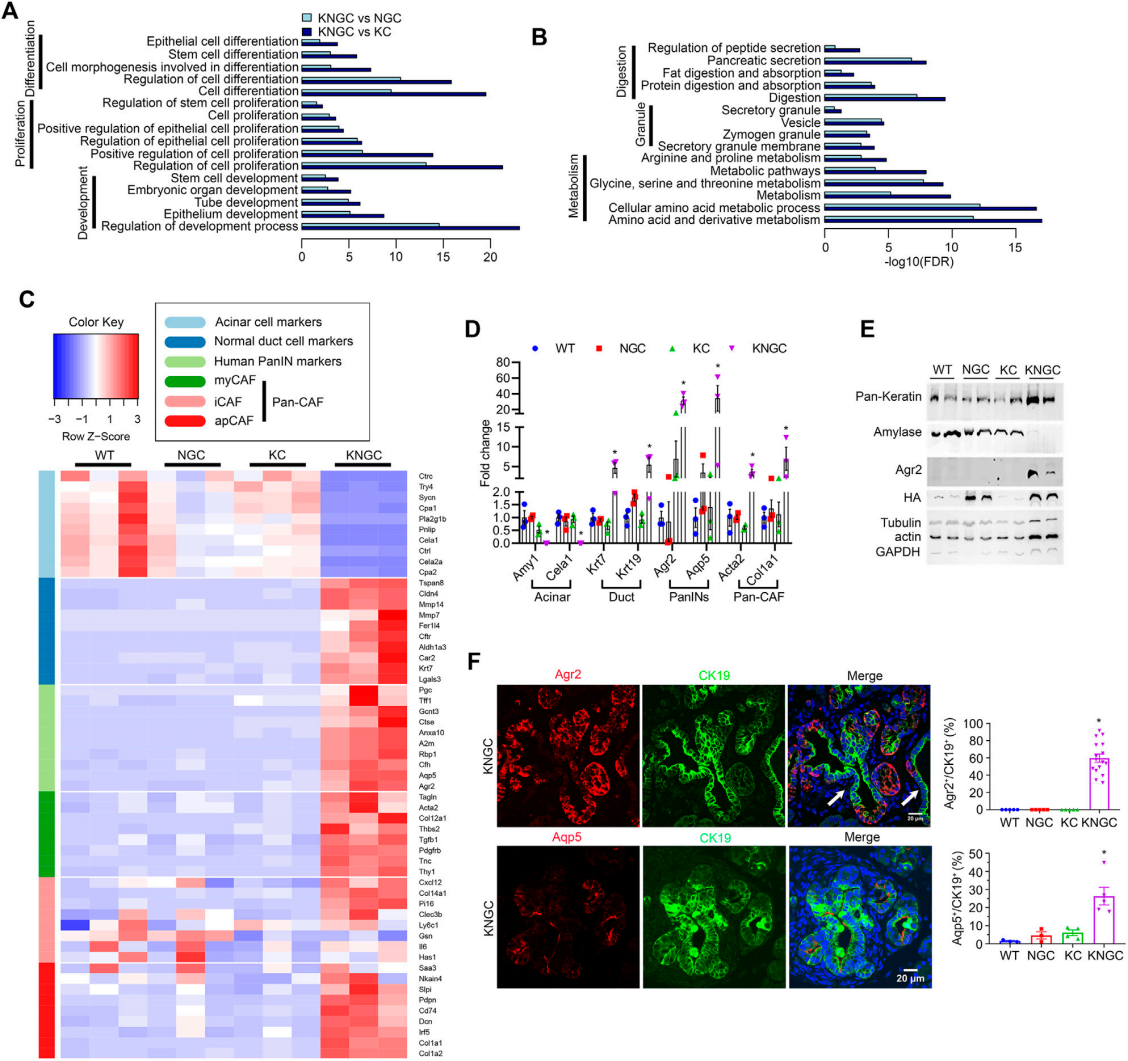
Transcriptional regulation of pancreatic ductal neoplasia by nuclear GSK-3β and Kras^G12D^. **(A)** KEGG cellular processes enriched for genes with increased expression in KNGC mice compared with NGC or KC. **(B)** KEGG cellular processes enriched for genes with decreased expression in KNGC mice compared with NGC or KC. **(C)** Heatmap was generated for selected gene sets related to the indicated groups using normalized gene expression. Colors are assigned based on raw z-scores. **(D)** Real-time PCR quantification of the indicated genes from 4-week-old WT, NGC, KC or KNGC mice. Data were analyzed and expressed as mean ± SEM. *n* = 3. ∗*p* < 0.05 KNGC mice versus the other genotypes. **(E)** Cell lysates from pancreas of 4-week-old WT, NGC, KC and KNGC mice were prepared and probed with the indicated antibodies. Shown are representative results from three experiments. **(F)** Immunofluorescence staining of Agr2 (red, top), Aqp5 (red, bottom) and CK19 (green) from pancreatic sections of 4-week-old KNGC mice. White arrow indicated Agr2^−^ (top) and Aqp5^−^ (bottom)/CK19^+^ cells. And quantification of Agr2^+^ and Aqp5^+^ percentage in CK19 cells was analyzed and expressed as mean ± SEM. ∗*p* < 0.05 KNGC mice versus the other genotypes.

To gain insight into the putative transcriptional pathways promoting the ductal hyperplasia seen in KNGC mice, we isolated RNA from the pancreas of 4-week-old WT, NGC, KC and KNGC mice and performed RNA-Seq. Consistent with the unusual composition of the pancreas in KNGC mice, we detected over 9,000 significantly changed genes compared to the other genotypes (Supplementary Figure S4A). KEGG signaling pathway analysis found over 1,000 pathways to be significantly activated, while 100 pathways were substantially inactivated in KNGC mice as compared to KC or NGC mice (using log2FC > 2 or log2FC < −2 as a cutoff, respectively; Supplementary Figure S4B). Among the signaling pathways increased in KNGC mice were those involved in differentiation, proliferation, and development (Figure 2A). In contrast, genes with decreased expression were closely associated with secretion and digestion, granule formation and cellular metabolism (Figure 2B). As can been seen in Figure 2C, markers for acinar cells, normal ductal cells, human PDA precursors, and multiple subsets of pancreatic cancer associated fibroblasts (Pan-CAF) (Reichert et al., 2013; Hoang et al., 2016; Muraro et al., 2016; Elyada et al., 2019), were significantly changed in KNGC mice compared to the other genotypes (complete list can be found in Supplementary Table S3). We next validated the RNA-Seq data *via* quantitative PCR for two genes from each compartment (Figure 2D). In addition, significantly increased protein levels of pan-keratin and Agr2, together with diminished expression of amylase, were confirmed by immunoblot (Figure 2E). Moreover, immunofluorescent staining of pancreata from KNGC mice with CK19 and amylase showed that the acinar tissue had been largely replaced with CK19^+^ neoplastic ducts (Supplementary Figures S5A,B).

Further analysis of our RNA-seq data indicated that the water channel Aquaporin 5 (Aqp5) had the highest log2FC among the 19 significantly increased genes compared between both NGC and KC with WT mice (Supplementary Figures S4C,D; detailed gene list can be found in Supplementary Table S4). Therefore, we investigated the expression of Agr2 and Aqp5 in the pancreas of KNGC mice. KNGC mice showed strong Agr2 expression in close to 60% of CK19^+^ ducts, while similarly aged KC mice only expressed Agr2 in a limited number of metaplastic ducts. Like Agr2 staining, Aqp5 was detected in a few early ADM lesions of KC mice but had significantly higher distribution in CK19^+^ ducts in KNGC mice (Figure 2F; Supplementary Figure S5C). Taken together, these data suggest that expression of GSK-3β and KRas^G12D^ result in a transcriptional switch from pancreatic acinar cells to ductal cells, with expression of pancreatic tumor precursor markers.

### Ductal Cells From KNGC Mice Express Genes Identified in Both Murine and Human Pancreatic Ductal Progenitors

**FIGURE 3:**
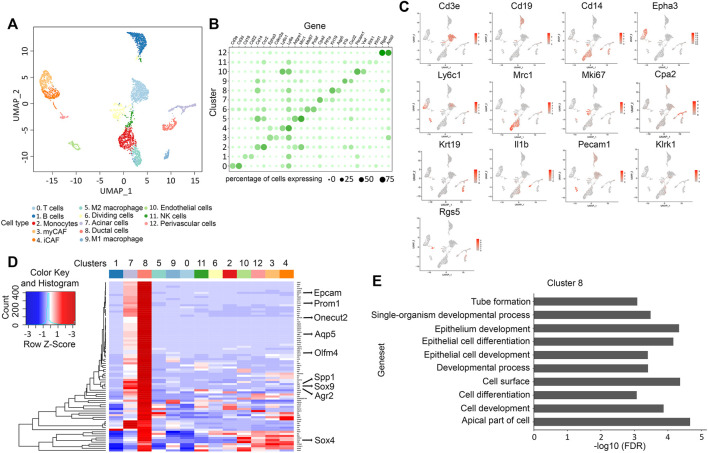
scRNA-Seq analysis reveals a progenitor-like ducts for the ductal hyperplasia in KNGC mice. **(A)** UMAP plot of 4,705 cells, colored by cell type identified by cluster markers. **(B)** Gene expression dot plot showing selected cluster-specific genes. Rows are clusters and columns are genes. Size of the dots indicates percentage of cells expressing a gene in each cluster. Color depth of the dot indicates normalized expression value. **(C)** UMAP plot of 4,705 cells colored by expression of selected genes from **(B)**. **(D)** Heatmap showing average expression of selected genes with log foldchange over 1 (lfc > 1) across all clusters. **(E)** Top over-represented pathways for marker genes of cluster 8.

To comprehensively catalog the cell populations represented in the KNGC pancreas, we conducted scRNA-seq to transcriptionally characterize over 6,000 equally mixed single cells from three 4-week-old KNGC mice. Using principal component analysis and clustering with the Seurat version 3.2.3 (Stuart et al., 2019), a uniform manifold approximation and projection (UMAP) plot of the cell suspension was generated, and 13 distinct clusters were identified by Leiden community detection algorithm (Traag et al., 2019) (Figure 3A). Signature genes within each cluster were cross-referenced with known markers of cell types from the literature to identify the different cell types that are represented by the clusters (Figures 3B,C). To our surprise, a substantial portion of the cells within the sequenced suspension were classified as immune cells, including B cells, T cells, NK cells, monocytes, and macrophages (Figure 3A) (Schlesinger et al., 2020). In addition, distinct populations of fibroblasts including myCAF (Epha3^+^) and iCAF (Ly6c1^+^) were also detected (Figures 3A–C) (Elyada et al., 2019). Although the acinar cell compartment is significantly reduced in KNGC mice as compared to the other genotypes (Figure 2C), we were able to capture acinar cells based on their gene expression signature (Figures 3A–C). Ductal cells, represented by cluster 8, were identified by the expression of Epcam, Krt7, Krt18, and Krt19 (Figure 3D). In addition, Aqp5 was detected as one of the most highly expressed genes in this cluster along with Agr2 and other previously identified progenitor cell markers, such as Spp1, Sox9, Sox4, Prom1, Olfm4 and Onecut2 (Figure 3D, full list of differential expressed genes in cluster 8 can be found in Supplementary Table S5) (Li et al., 2007; Lynn et al., 2007; Oshima et al., 2007; Jin et al., 2016; Yu et al., 2019; Qadir et al., 2020). Consistent with the recently identified role of Aqp5 in mouse and human adult pyloric stem cells (Tan et al., 2020), gene expression and gene set enrichment analysis (GSEA) revealed activation of signaling pathways associated with cell differentiation and development (Figure 3E). These data suggest that expression of nuclear-targeted GSK-3β and KRas^G12D^ during early pancreatic development promotes the retention of an Aqp5^+^ ductal population with progenitor-like characteristics.

### Expression of Nuclear GSK-3β With KRas^G12D^ Leads to the Development of Two Distinct Ductal Populations

**FIGURE 4:**
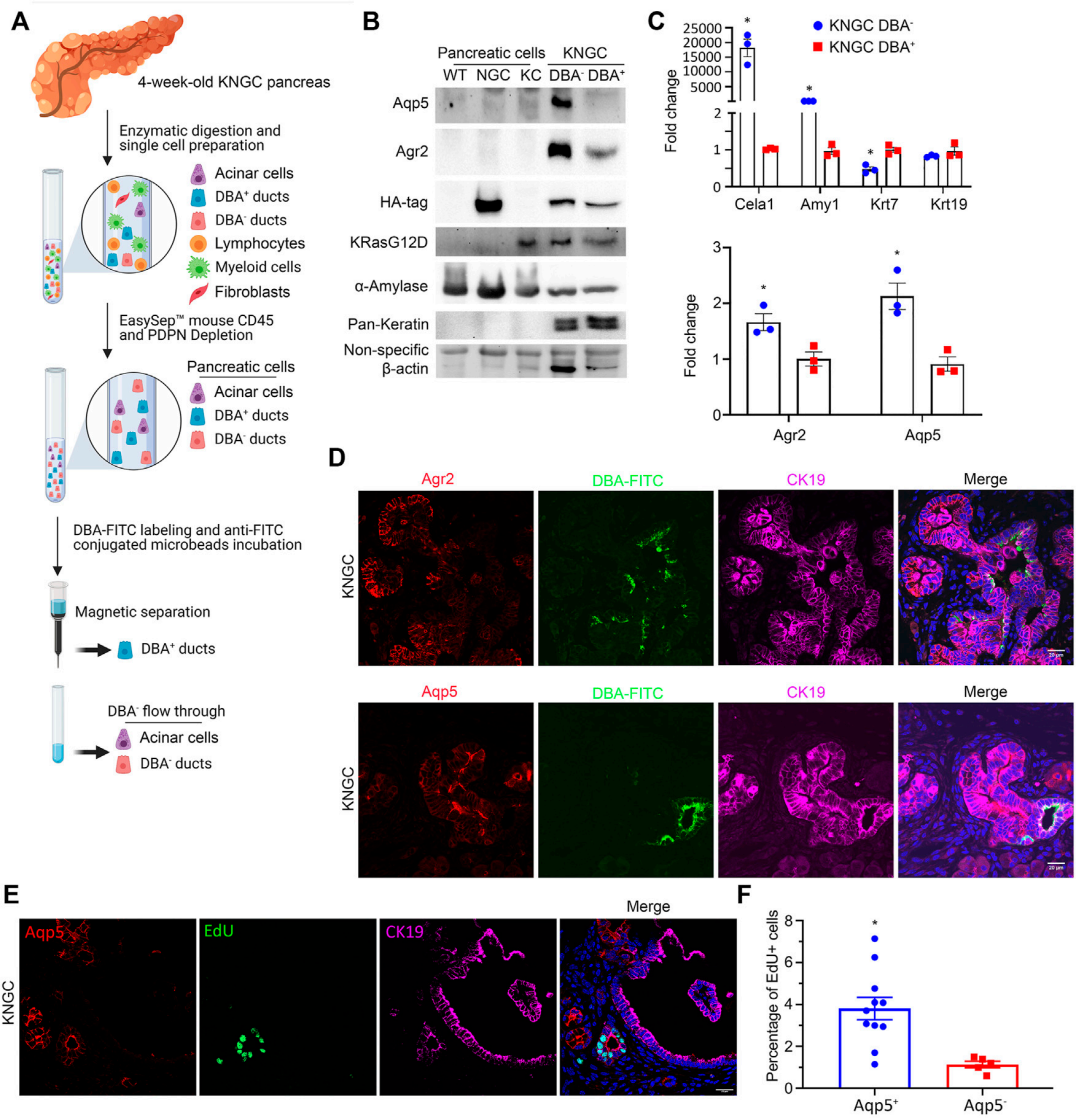
KNGC mice develop two distinct ductal populations. **(A)** Illustration of the experimental approach to purify lectin-expressing ductal cells through negative selection (anti-CD45 and anti-Podoplanin) and purification using the lectin-binding protein DBA. **(B)** Cell lysates from isolated DBA^−^ and DBA^+^ cell pools of five 4-week-old KNGC mice and pancreatic cells from WT, NGC, KC mice were prepared and probed with the indicated antibodies. **(C)** Real-time PCR quantification of the indicated genes from isolated DBA^−^ and DBA^+^ samples from 4-week-old KNGC mice. TBP, β-actin, RPLP0 and GAPDH were used as internal housekeeping gene controls. Data were analyzed and expressed as mean ± SEM. *n* = 3; ∗*p* < 0.05. **(D)** Immunofluorescence staining of Agr2 (red, upper panel) or Aqp5 (red, lower panel) with DBA-FITC (green) from pancreatic sections of 4-week-old KNGC mice. **(E)** Immunofluorescence staining of EdU (green), Aqp5 (red) and CK19 (purple) from pancreatic sections of 4-week-old KNGC mice. **(F)** Quantification of EdU positive percentage in Aqp5 positive or negative cells was analyzed and expressed as mean ± SEM. ∗*p* < 0.05 EdU positive in Aqp5 positive versus negative cells.

To further characterize the neoplastic ductal cells arising in the KNGC model, we utilized a modified protocol to capture ductal cells from the pancreas using the lectin Dolichos Bifluros Agglutin (DBA) that binds to α-linked N-acetyl-D-galactosamine (GalNAc) present on pancreatic ductal cells (Reichert et al., 2013). Single cell suspension of pancreatic cells comprised of acinar and ductal cells were enriched by depleting immune cells and fibroblasts using anti-CD45 and anti-podoplanin (PDPN) antibodies, respectively (Auciello et al., 2019; Elyada et al., 2019; Helms et al., 2020). The collected pancreatic cells were then incubated with DBA to capture GalNAc^+^ ductal cells with magnetic beads. This should result in the separation of a DBA^+^/CK19^+^ ductal cell pool from a DBA^−^/CK19^−^ cell population comprised mainly of acinar cells. Fluorescence microscopy confirmed the capture of DBA-FITC-labeled cells from 4-week-old KNGC mice (Supplementary Figure S6A). Notably, there were very few DBA^+^/CK19^+^ ductal cells eluted in WT, NGC, and KC mice of similar age (Supplementary Figure S6B), so pancreatic cells from those genotypes were collected as control without DBA purification. To our surprise, and as shown in Figure 4A, we observed equivalent HA expression in both the DBA^−^ and DBA^+^ samples, with significantly higher levels of Aqp5 and Agr2 but lower levels of pan-keratin in the DBA^−^ pool compared to the DBA^+^ ductal pool (Figure 4B). Using qRT-PCR, we found that DBA^−^ cells from 4-week-old KNGC mice exhibited higher acinar cell marker expression and reduced ductal marker expression compared to the DBA^+^ population. More importantly, the DBA^−^ population from 4-week-old KNGC mice had increased expression of Agr2 and Aqp5 (Figure 4C). These results indicate that the cell population expanding in KNGC mice represents ductal population harboring progenitor cell features but lacking the glycans that are bound by DBA.

We next examined the differential expression of Agr2 and Aqp5, along with DBA, in neoplastic ducts from KNGC mice using immunofluorescence. Co-staining of Agr2, Aqp5 and DBA-FITC showed strongly positive Agr2 and Aqp5 stained ducts that were distinct from those detected using DBA-FITC (Figure 4D). Strikingly, EdU labeling showed increased DNA synthesis in Aqp5^+^ ductal cells compared to DBA-FITC^+^ ductal cells in KNGC mice (Figures 4E,F). Taken together, these data suggest that GSK-3β and KRas^G12D^ lead to the development of an DBA^−^/Aqp5^+^ ductal cell pool with proliferative potential.

### Aqp5 Is Necessary for the Differentiation and Growth of Stem-Like Ducts in KNGC Mice

**FIGURE 5:**
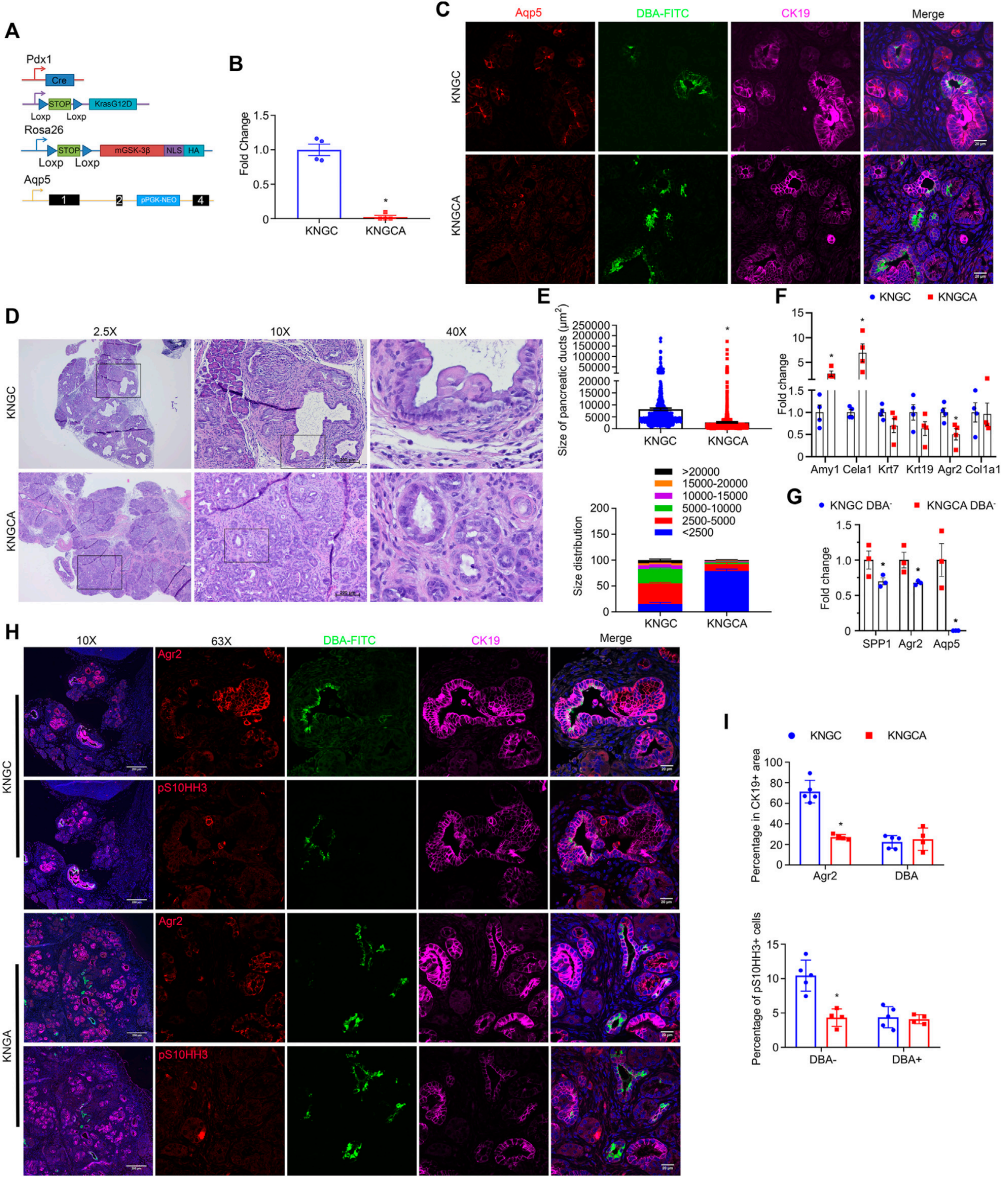
Aqp5 is necessary for the differentiation and growth of terminal ducts in KNGC mice. **(A)** Schematic representation of KNGCA (LSL-KRas^G12D^/Rosa26-LSL-nuclear GSK-3β/Pdx1-Cre/Aqp5 knockout) mouse model. Blue triangles indicate loxP sites. Black rectangles indicate exons of *aqp5* gene. **(B)** Real-time PCR quantification of Aqp5 gene expression from 4-week-old KNGC and KNGCA mice. TBP, β-actin, RPLP0 and GAPDH were used as internal housekeeping gene controls. Data were analyzed and expressed as mean ± SEM. ∗*p* < 0.05 KNGCA versus KNGC mice. **(C)** Immunofluorescence staining of Aqp5 (red), DBA-FITC (green) and CK19 (purple) from pancreatic sections of 4-week-old KNGC and KNGCA mice. **(D)** H&E-stained pancreatic sections from KNGC and KNGCA mice. Black boxes indicated area magnified. Bars = 200 μm. **(E)** Quantification of pancreatic ducts size distribution and average pancreatic duct size were analyzed and expressed as mean ± SEM. *n* = 3. ∗*p* < 0.05 KNGCA versus KNGC mice. **(F)** Real-time PCR quantification of the indicated genes expressions from 4-week-old KNGC and KNGCA mice. Data were analyzed and expressed as mean ± SEM. ∗*p* < 0.05 KNGCA versus KNGC mice. **(G)** Real-time PCR quantification of the indicated gene expression in DBA^−^ ducts from 4-week-old KNGC and KNGCA mice. Data were analyzed and expressed as mean ± SEM. ∗*p* < 0.05 KNGCA versus KNGC mice. **(H)** Immunofluorescence staining of Agr2 (red, upper panel) or pS10HH3 (red, lower panel) with DBA-FITC (green) and CK19 (purple) from serial pancreatic sections of 4-week-old KNGC and KNGCA mice. **(I)** Quantification of percentage in CK19 positive cells, as well as percentage of pS10HH3 positive cells in DBA^−^ and DBA^+^ ducts were analyzed and expressed as mean ± SEM. ∗*p* < 0.05 KNGCA versus KNGC mice.

The water channel Aqp5 was first described to be localized mainly in terminal/intercalated and interlobular ducts in the human pancreas (Burghardt et al., 2003; Méndez-Giménez et al., 2018), which are also characterized as DBA^−^ (Nakano et al., 2015). A recent study also identified Aqp5 expression in gastric stem cells (Tan et al., 2020). To determine the contribution of Aqp5 to the development and growth of neoplastic ducts, we crossed KNGC mice with a whole body Aqp5 knockout (Krane et al., 2001) to generate KNGCA mice lacking Aqp5 expression (Figures 5A–C). Histological examination revealed that the knockout of Aqp5 in KNGC mice significantly decreased the development of neoplastic ducts, when compared to similarly aged KNGC mice (Figures 5D,E). In addition, expression of acinar markers was restored in KNGCA mice (Figure 5F; Supplementary Figures S7A,B). Moreover, expression of Agr2 and Spp1 were reduced from whole tissue and isolated DBA^−^ cells from KNGCA mice compared to KNGC mice (Figures 5F,G). To further characterize the impact of Aqp5 deletion on the development of Agr2^+^ ductal cells and proliferation, serial sections of pancreas from KNGC and KNGCA mice were stained using a combination of Agr2 or pS10HH3 with DBA-FITC. As shown in Figure 5H, pS10HH3, mainly localized in DBA^−^/Agr2^+^ ducts in KNGC mice, whereas KNGCA mice showed fewer pS10HH3^+^ DBA^−^ cells, as well as a reduction in the percentage of Agr2^+^ cells (Figure 5I). Moreover, KNGCA mice showed a higher percentage of acinar cells and smaller size and area of ductal cells than KNGC mice at 6–8 months of age (Supplementary Figures S7C,D). These data reveal an important role for Aqp5 in the development and proliferation of the Agr2^+^ ductal population in KNGC mice.

### Human IPMN Samples Contain Aqp5, Agr2 and GSK-3β Positive Ducts

**FIGURE 6:**
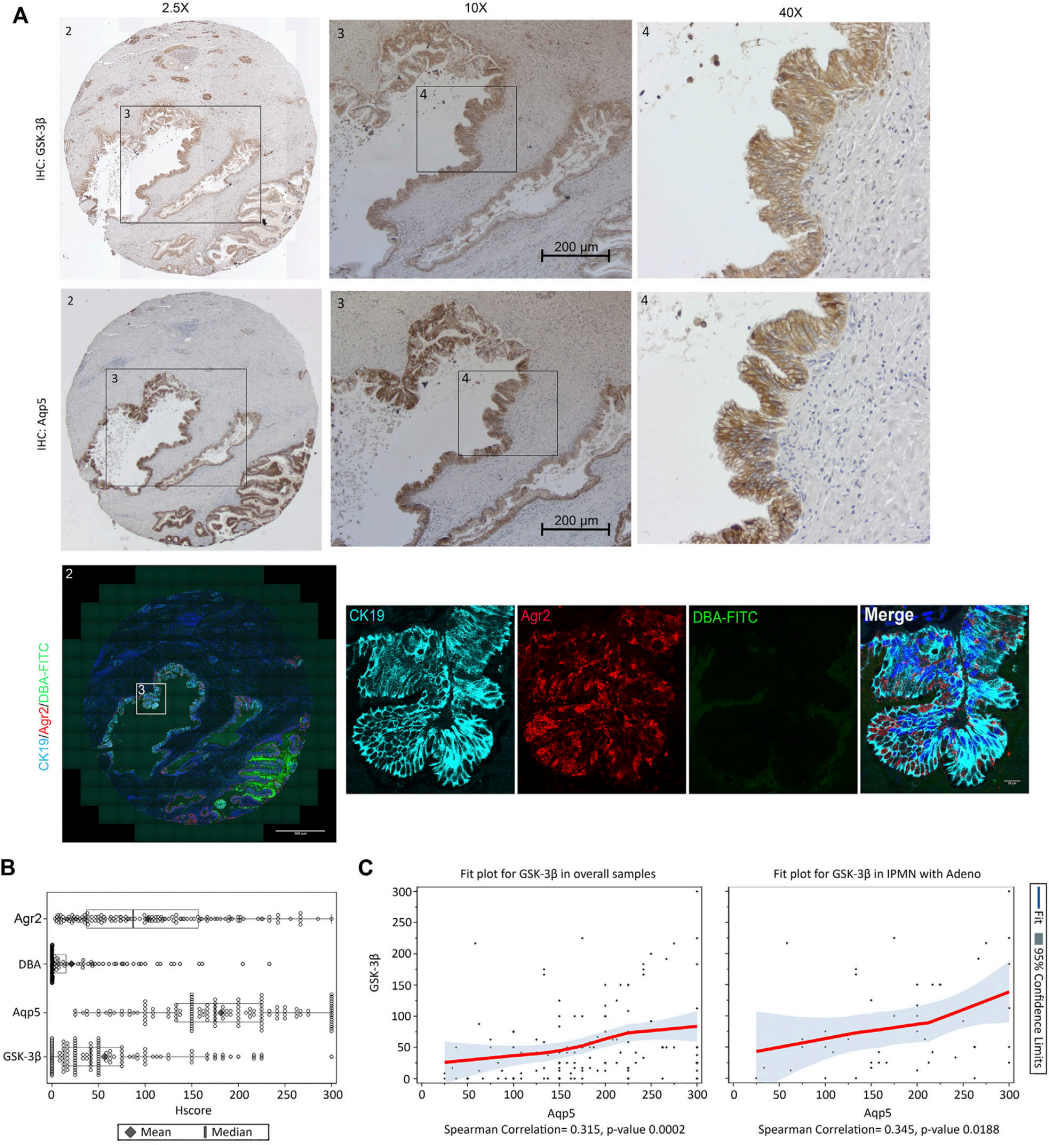
Aqp5, GSK-3β and Agr2 but not DBA staining is enriched in human IPMN. **(A)** Representative immunohistochemistry staining of GSK-3β (Top), Aqp5 (Middle), and immunofluorescence staining of Agr2 (red) with DBA-FITC (green) and CK19 (cyan) (Bottom) from serial sections of the same human IPMN sample. Bars = 200 μm. **(B)** Histological score (Hscore) of Aqp5, GSK-3β, Agr2 and DBA staining in 140 samples of human IPMN were calculated and graphed. The mean ± SD is shown. Black diamond: Mean value. Black line: Median value. **(C)** Scatter plot with loess fit line and 95% confidence limits (colored area) for Aqp5 and GSK-3β in overall samples (left) and IPMN with adenocarcinoma (right) were drawn and Spearman correlation coefficient were calculated with *p*-value.

To examine whether Aqp5^+^/Agr2^+^/DBA^−^ ductal cells were associated with IPMN development in an established mouse model for IPMN, we stained Aqp5, Agr2 and DBA in sections obtained from KGC mice, which combines KRas^G12D^ and GNAS mutation (Patra et al., 2018). Significantly, the ductal lesions from KGC mice had higher staining for both Aqp5 and Agr2 than DBA-FITC (Supplementary Figures S8A,B). To determine if this was relevant to human IPMN, we performed Agr2/DBA-FITC IF staining as well as Aqp5 and GSK-3β IHC staining on serial sections from a tissue microarray (TMA) containing 140 human IPMN cases (see patient demographics of TMA in Supplementary Table S6) (Whitcomb et al., 2012). Consistent with our KNGC mouse model, the majority of IPMN cores showed positive staining for Aqp5, Agr2 and GSK-3β and significantly fewer cores that were DBA^+^ (Core 1 and 2 in Figure 6A; Supplementary Figure S9A). The strong and widespread distribution of Agr2^+^/Aqp5^+^/DBA^−^ ductal cells in the IPMN TMA were further confirmed by Hscore, as Agr2 and Aqp5 staining were much higher than the DBA-FITC staining (Figure 6B). Moreover, correlation analysis of Aqp5 and GSK-3β using Hscore indicated a statistically significant correlation between Aqp5 and GSK-3β in the overall samples and in the subset of IPMN with adenocarcinoma (Figure 6C). Collectively, these results support the idea that an Aqp5^+^ ductal cell might contribute to the initiation and development of mouse and human IPMN.

## Discussion

In this study, we generated a new mouse model to investigate the contribution of nuclear GSK-3β to KRas^G12D^-driven pancreatic cancer development. Surprisingly, we found that 4-week-old KNGC mice develop pancreatic cysts and metaplastic duct cell expansion. In a longitudinal study, KNGC mice show a notable reduction of body weight, as well as shortened overall survival, which is likely do to the loss of pancreatic acinar cells and overall pancreatic physiology. RNA-seq of the whole pancreas identified robust expression of Aqp5 and Agr2, which have been identified in PDA precursors (Hezel et al., 2006; Reichert et al., 2013; Zhang et al., 2021; Ma et al., 2022). Taking advantage of scRNA-seq, we were able to confirm the dominant expression of these genes in the ductal compartment and further uncover an expression profile reflecting features of pancreatic ductal progenitor cells. Consistent with previous publications (Burghardt et al., 2003; Nakano et al., 2015; Dumartin et al., 2017; Tan et al., 2020), these progenitor-like ductal cells possess a higher proliferative potential than DBA-lectin^+^ well-differentiated ducts (Figure 4E). Significantly, knockout of Aqp5 in KNGC mice impaired IPMN development, indicating that Aqp5 is likely involved in pancreatic progenitor-like duct cell retention/proliferation. We also observed the existence of widespread Aqp5^+^/Agr2^+^/DBA^−^ ducts in human IPMN samples, and in a mouse model for IPMN (Patra et al., 2018). Taken together, these results suggest that Aqp5^+^ pancreatic duct cells could contribute to human IPMN development.

Glycogen synthase kinases, GSK-3α and GSK-3β, are involved in a variety of cellular processes and the influence of GSK-3 kinases to those cellular processes is largely dictated by their direct or indirect regulation of protein targets (Cormier and Woodgett, 2017). Thus, it is not surprising that GSK-3 kinases may have paradoxical roles in seemingly similar cellular process in a context-dependent manner. For example, genetic ablation or pharmacological inhibition of GSK-3β has been shown to promote the proliferative capacity and self-renewal of embryonic stem cells and low level of GSK-3 activity could promote proliferation in neural progenitor cells (NPC’s) through transcription regulators like c-myc, Klf5 and Wnt/β-catenin pathway (Sato et al., 2004; Jiang et al., 2008; Ying et al., 2008; Bone et al., 2009; Eom and Jope, 2009; Hall et al., 2009; Kim et al., 2009; Mao et al., 2009). In contrast, nuclear GSK-3β is not only responsible for cancer stem cell self-renewal and glioblastoma tumorigenesis by stabilizing histone demethylase KDM1A but also crucial for acute myeloid leukemia (AML) initiation and aggressiveness in AML *via* β-catenin independent mechanisms (Wang et al., 2008; Wang et al., 2010; Gupta et al., 2012; Zhou et al., 2016; Ignatz-Hoover et al., 2018). Although a negative regulatory role for GSK-3β in pancreatic β-cell proliferation and development was shown (Liu et al., 2008; Tanabe et al., 2008; Liu et al., 2010), we and others found that GSK-3β regulates KRas^G12D^-induced pancreatic acinar-to-ductal metaplasia (ADM) and proliferation by increasing the activation of S6 kinase (Shin et al., 2011; Ding et al., 2017), suggesting a cooperation between KRas^G12D^ and GSK-3β signaling pathways in promoting proliferation in the ductal cell lineage. Moreover, we show here that RKO mice have decreased accumulation of all PanIN precursor lesions and invasive cancer compared to similarly aged KC mice, further highlighting its importance in PDA precursor development and progression. While it is not clear what leads to the nuclear accumulation of GSK-3β we have previously observed in human PDA (Ougolkov et al., 2006), our data shown here would suggest that nuclear GSK-3β (comparing NGC to KNGC mice) alone cannot promote progenitor ductal cell retention but requires the cooperation of KRas^G12D^ signaling pathways that remain to be elucidated.

Numerous studies using rodent and human pancreas have identified a potential pool of pancreatic ductal progenitors (Cornelius et al., 1997; Ramiya et al., 2000; Suzuki et al., 2002; Inada et al., 2008; Xu et al., 2008). While flow cytometry has been adapted to isolating these ductal progenitors using surface markers (Suzuki et al., 2004; Rovira et al., 2010; Qadir et al., 2018), the *in vivo* localization and molecular characteristics of this cell population remain largely unknown due to their limited numbers in the adult mouse pancreas (Kuise et al., 2013). Using embryonic tissues and scRNA-seq, cell populations in mouse pancreas have been identified that give rise to both endocrine and exocrine cell types, as well as those that initiate the ductal cell lineage (Yu et al., 2019). Similarly, scRNA-seq identified subgroups of pancreatic ductal progenitor cells in the human pancreas that favor acinar or ductal cell differentiation (Qadir et al., 2018). In agreement with the above studies, our scRNA-seq data showed expression of markers like Epcam, Onecut2, Sox9, Sox4, Spp1, Olfm4, and Prom1 in Aqp5^+^ ductal cells. Consistent with the role of Aqp5 as a source for early tumor formation in gastric cancer (Tan et al., 2020) and pancreas injury-associated pancreatic precursor initiation (Ma et al., 2022), Aqp5^+^ progenitor-like ductal cells from KNGC mice are indeed more proliferative and deletion of Aqp5 in KNGC animals results in more acinar cell development and less ductal formation. Although it is not clear how Aqp5 sustains this ductal progenitor cell pool, our data suggest that activation of nuclear GSK-3β and KRas^G12D^ within this pool of cells drives their retention and ultimately the development of IPMN lesions.

In summary, our study provides evidence that nuclear GSK-3β and oncogenic KRas^G12D^ signaling cooperate to promote the retention of an Aqp5^+^ ductal cell population with progenitor-like characteristics that can also be found in human and mouse IPMN. Further characterization of the KNGC mouse model has the potential to not only identify the signaling pathways and transcriptional networks that facilitate the development of the ductal cell compartment in the pancreas but could aid in the development of biomarkers to detect pre-cancerous ductal lesions in the pancreas.

## Data Availability

The datasets presented in this study can be found in online repositories. The names of the repository/repositories and accession number(s) can be found below: https://www.ncbi.nlm.nih.gov/geo/, GSE169618, GSE153548.
