# Supplementary material for: Nuclear GSK-3β and Oncogenic KRas Lead to the Retention of Pancreatic Ductal Progenitor Cells Phenotypically Similar to Those Seen in IPMN
Source: Front Cell Dev Biol. 2022 May 13;10:853003. doi: 10.3389/fcell.2022.853003 (PMC9136019; doi:10.3389/fcell.2022.853003)
Supplement: Supplementary file 1 [file DataSheet2.PDF]

Supplemental Material and Methods

RNA isolation and quantitative RT-PCR

RNA isolation and quantitative RT-PCR were performed as previously described [1]. Briefly, pancreatic total RNA or isolated pancreatic ductal cells was isolated using Trizol and further purified with a RNeasy Mini Kit (Qiagen, Valencia, CA). Reverse transcription was performed with the Superscript III RT-PCR Kit (Invitrogen). Quantitative PCR was performed with the SYBR Green PCR Master Mix using the ABI StepOnePlus Sequence Detection System (Applied Biosystems, Carlsbad, CA). TBP, β-actin, RPLP0 and GAPDH were used as internal housekeeping gene controls for normalization of gene expression. The double Δ Ct method was used to analyze gene expression. Experiments were performed a minimum of three times using independent cDNAs. Primer sequences are provided in Supplemental Excel Table S2.

RNA-Seq and data analysis

Total RNA was isolated from 4 weeks old littermates as described above and the average RNA integrity number values were measured by an Agilent Bioanalyzer. Individual transcriptome sequencing (RNA-Seq) libraries were prepared from each mouse using an Illumina TruSeq v2 kit. 100 base pairs reads were collected with an Illumina HiSeq 4000 instrument. Fastq files of paired-end reads were aligned with STAR 2.6.0a [2] to the UCSC reference genome mm10 with basic 2-pass mapping. Gene counts were obtained using the subRead Feature Counts program 1.4.6 [3] based on the UCSC mm10 annotation. Differential expression analyses were performed using R package DESeq2 1.10.1 [4] after removing genes with average raw counts less than 10.

Genes with log2 fold change greater than 2 or less than -2, and FDR less than 0.05 were considered significantly differentially expressed. For functional annotation analysis, the Fisher's exact test was used to determine overrepresented pathways or gene sets in significantly up- or down-regulated genes, against gene sets described in Enrichment Map [5] and R package KEGG.db [6]. RNA sequencing data have been deposited in the Gene Expression Omnibus under the accession number GSE153548.

Single-Cell Library Preparation and RNA-seq Data Processing

Preparation of single-cell suspensions from the mouse pancreas were performed as described above. Isolated cells were washed and resuspended in PBS containing 0.04% BSA. Cells were then counted on Vi-Cell XR Cell Viability Analyzer (Beckman-Coulter), and up to 6000 cells were loaded per lane on an 10X Genomics Chromium system. Single-cell capture, barcoding, and library preparation were performed using the 10X Genomics Chromium system according to the manufacturer's protocol. cDNA and libraries were checked for quality on Qubit High Sensitivity assays (Thermo Fisher Scientific), Agilent Bioanalyzer High Sensitivity chips (Agilent) and quantified by Kapa DNA Quantification reagents (Kapa Biosystems) before sequencing 60,000 fragment reads per cell following Illumina's standard protocol using the Illumina cBot and HiSeq 3000/4000 PE Cluster Kit. Single-cell RNA sequencing data have been deposited in the Gene Expression Omnibus under the accession number GSE169618.

Reads from the single-cell RNA-seq dataset in fastq format were mapped to the mm10 reference genome using Cellranger version 3.0.2 [7]. The gene expression by barcode matrix of the sequenced library was imported into Seurat version 3.2.3 [8]. Only genes

that are expressed in more than 3 cells were kept; while barcodes (cells) with more than 500 expressed genes, between 1,000 and 81,455 (three times the standard deviations above the mean) unique molecular identifier (UMI) and <0.5 mitochondrial gene content were retained. Raw counts were natural log-transformed and multiplied by a factor of 1000, then centered and scaled after regressing out the number of UMI of each cell. Principal component analysis (PCA) was calculated based on all genes present in the dataset and a scree plot was used to determine the number of principal components to use for downstream analysis. To cluster the cells a k-nearest neighbor (KNN) graph is first constructed based on Euclidean distance in PCA space using top 10 principal components. This KNN graph is then used to generate a shared nearest neighbor (SNN) graph by calculating the neighborhood overlap using the Jaccard index. A modularity optimization process using the Louvain algorithm is then applied to iteratively group cells together using a resolution parameter of 0.6, resulting in cell clusters. To place cells in two-dimensional space for visualization based on similarity, UMAP projection using the Leiden community detection algorithm [9] was performed on the same top 10 principal components and the results shown in scatter plots.

Genes that are highly expressed in each cluster, i.e. cluster markers were detected using FindAllMarkers function from the Seurat package by comparing each individual cluster against all other clusters in the dataset. The obtained gene lists were defined based on Wilcoxon rank-sum test with p-value < 0.05 and log2 fold change of > 0.25. Only genes detected in at least 25% of the cells within the given cluster were considered. The cluster markers were then compared to published cell type markers to identify cell types. Functional analysis was performed for each cluster marker gene lists

using the Fisher exact test for over-representation against the mouse gene ontology and pathway gene sets maintained in the Enrichment Map [5].

Reference

1. Ding L, Liou GY, Schmitt DM*, et al.* Glycogen synthase kinase-3beta ablation limits pancreatitis-induced acinar-to-ductal metaplasia. *J Pathol* 2017; **243**: 65-77.
2. Dobin A, Davis CA, Schlesinger F*, et al.* STAR: ultrafast universal RNA-seq aligner. *Bioinformatics* 2013; **29**: 15-21.
3. Liao Y, Smyth GK, Shi W. featureCounts: an efficient general purpose program for assigning sequence reads to genomic features. *Bioinformatics* 2014; **30**: 923-930.
4. Love MI, Huber W, Anders S. Moderated estimation of fold change and dispersion for RNA-seq data with DESeq2. *Genome Biol* 2014; **15**: 550.
5. Merico D, Isserlin R, Stueker O*, et al.* Enrichment map: a network-based method for gene-set enrichment visualization and interpretation. *PLoS One* 2010; **5**: e13984.
6. M C. KEGG.db: A set of annotation maps for KEGG. R package version 3.2.3. 2016.
7. Zheng GX, Terry JM, Belgrader P*, et al.* Massively parallel digital transcriptional profiling of single cells. *Nat Commun* 2017; **8**: 14049.
8. Stuart T, Butler A, Hoffman P*, et al.* Comprehensive Integration of Single-Cell Data. *Cell* 2019; **177**: 1888-1902 e1821.
9. Traag VA, Waltman L, van Eck NJ. From Louvain to Leiden: guaranteeing well-connected communities. *Sci Rep* 2019; **9**: 5233.
