# Supplementary material for: Nuclear GSK-3β and Oncogenic KRas Lead to the Retention of Pancreatic Ductal Progenitor Cells Phenotypically Similar to Those Seen in IPMN
Source: Front Cell Dev Biol. 2022 May 13;10:853003. doi: 10.3389/fcell.2022.853003 (PMC9136019; doi:10.3389/fcell.2022.853003)
Supplement: Supplementary file 2 [file DataSheet1.PDF]

Supplemental Excel File and Figures legend

Supplemental Excel File: Table S1 – Antibodies used in this study, **Table S2** – primers used for qPCR, **Table S3** – complete list of group markers (Figure 2), **Table S4** – commonly increased genes (Supplemental Figure S2), **Table S5** – complete list of differential expressed genes in Cluster 8 (Figure 3) and **Table S6** – patient demographics for TMA (Figure 6).

Li Ding_Supplemental FigS1

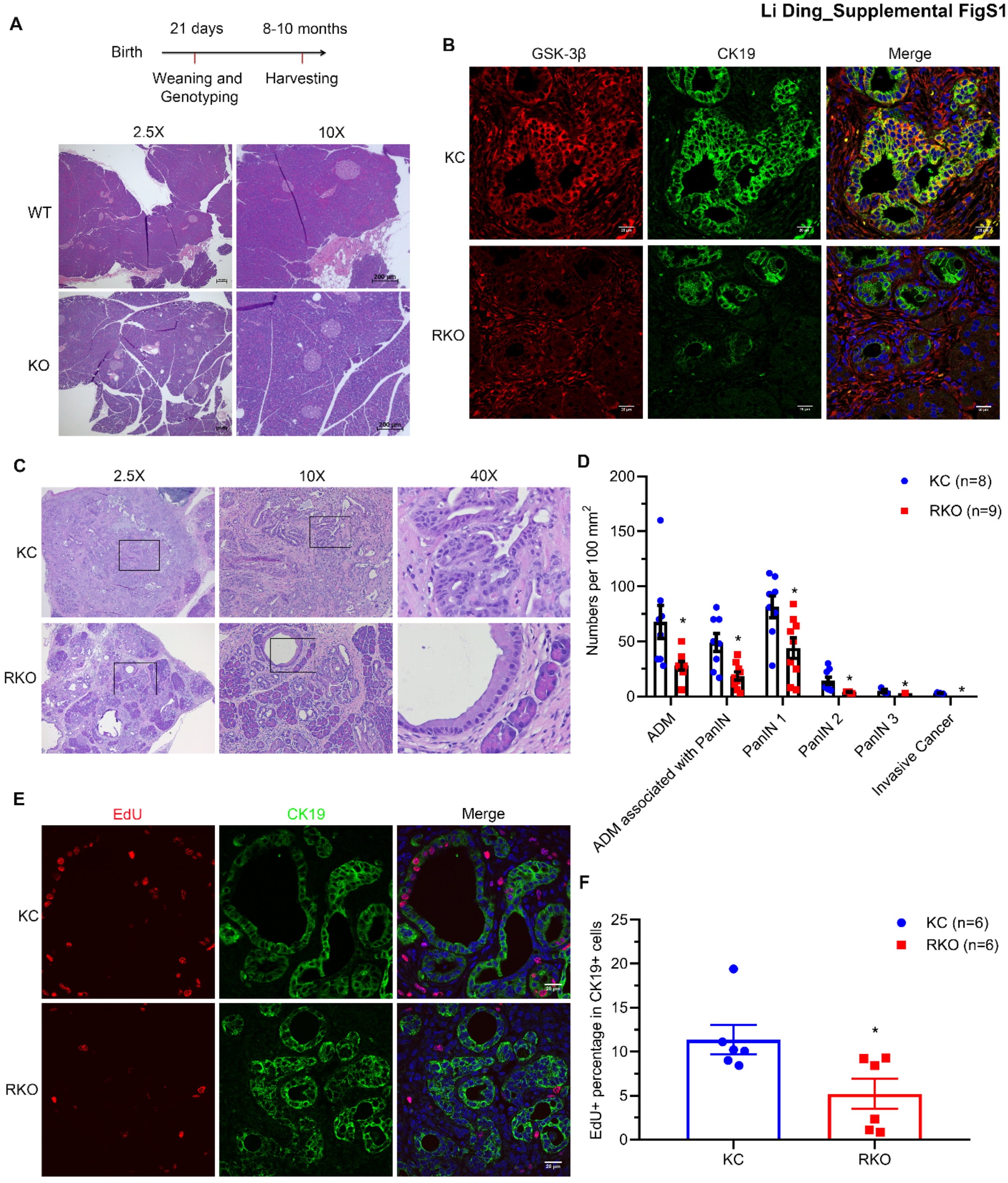

Figure S1. GSK-3β deletion limits $KRas^{G12D}$-induced pancreatic cancer development. (A) Scheme for aging-induced pancreatic cancer progression model and analysis (Upper panel). H&E-stained pancreatic sections from WT and KO mice (Lower panel). (B) Immunofluorescence staining of GSK-3β (red) and CK19 (green) from pancreatic sections of aging KC and RKO mice. (C) H&E-stained pancreatic sections from KC and RKO mice. Black boxes indicate magnified area. (D) H&E-stained tissue samples from KC and RKO mice were evaluated and quantitatively analyzed for numbers per 100 mm^2. Data were analyzed and expressed as mean ± SEM. *$P<0.05$ RKO versus KC mice. (E) Double labeling of pancreatic sections from aging KC and RKO mice was performed using incorporated EdU detection kit (red) and CK19 (green) antibodies. (F) Quantification of the percentage of EdU positive neoplastic ductal cells in aging KC and RKO mice. Data were analyzed and expressed as mean ± SEM. n = 6. *$P<0.05$ RKO versus KC mice.

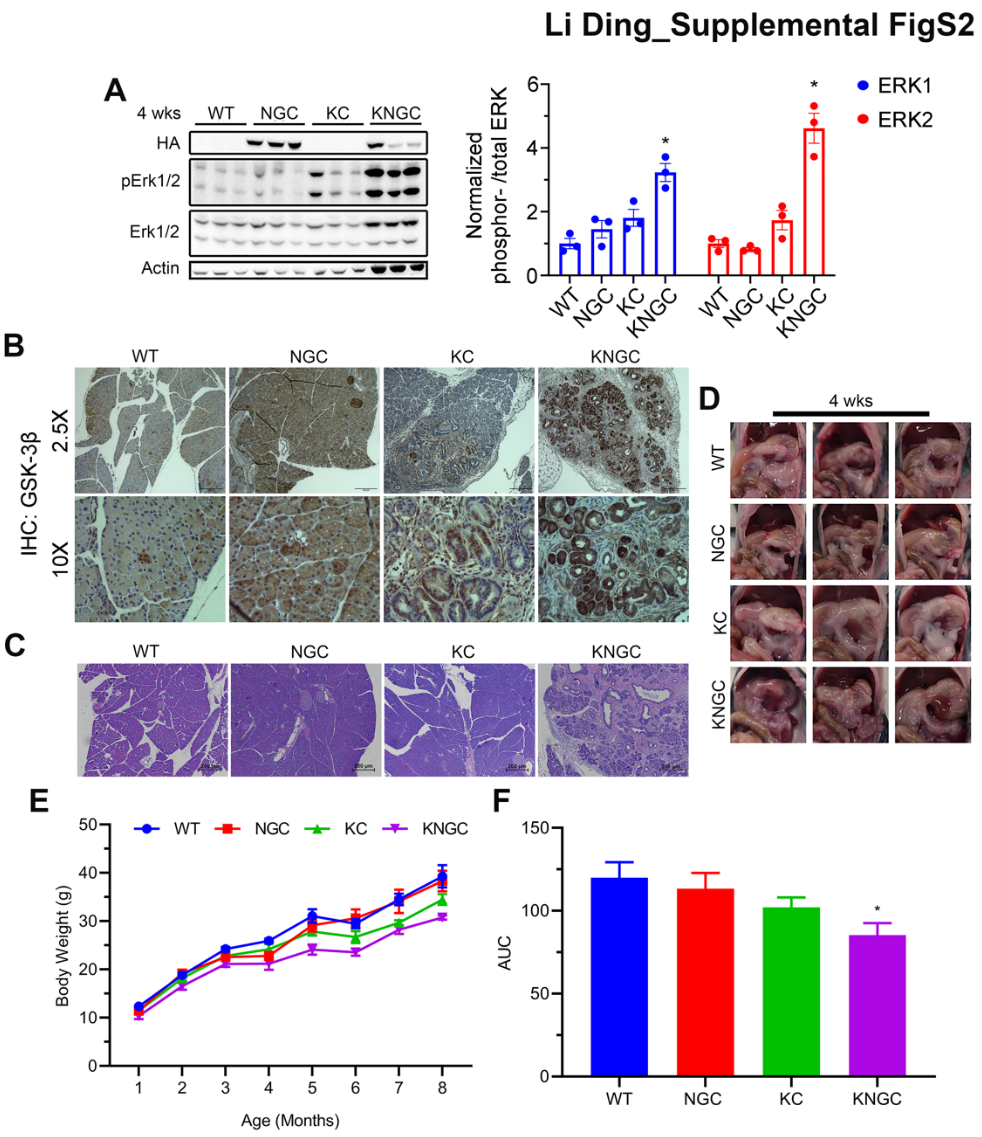

Figure S2. Nuclear GSK-3β and KRas^{G12D} promote pancreatic ductal cells expansion and IPMN development. (A) Cell lysates from the indicated genotypes at 4-week-old were prepared and probed with the indicated antibodies. Shown are representative results from 6 experiments. *P<0.05 KNGC mice versus the other genotypes. (B) and (C) Immunohistochemistry staining of GSK-3β and H&E-stained pancreatic sections from WT, NGC, KC and KNGC mice at 4-week-old. Bars = 200 μm. (D) Gross pathology of pancreas and adjacent tissues from transgenic mice of indicated genotypes at 4-week-

old. Body weight (E) and area under the curve (AUC) (F) of WT, NGC, KC and KNGC mice fed normal chow diet were measured at the indicated time points. Data were analyzed and expressed as mean ± SEM. At least 10 mice were included at each point. *P<0.05 KNGC mice versus the other genotypes.

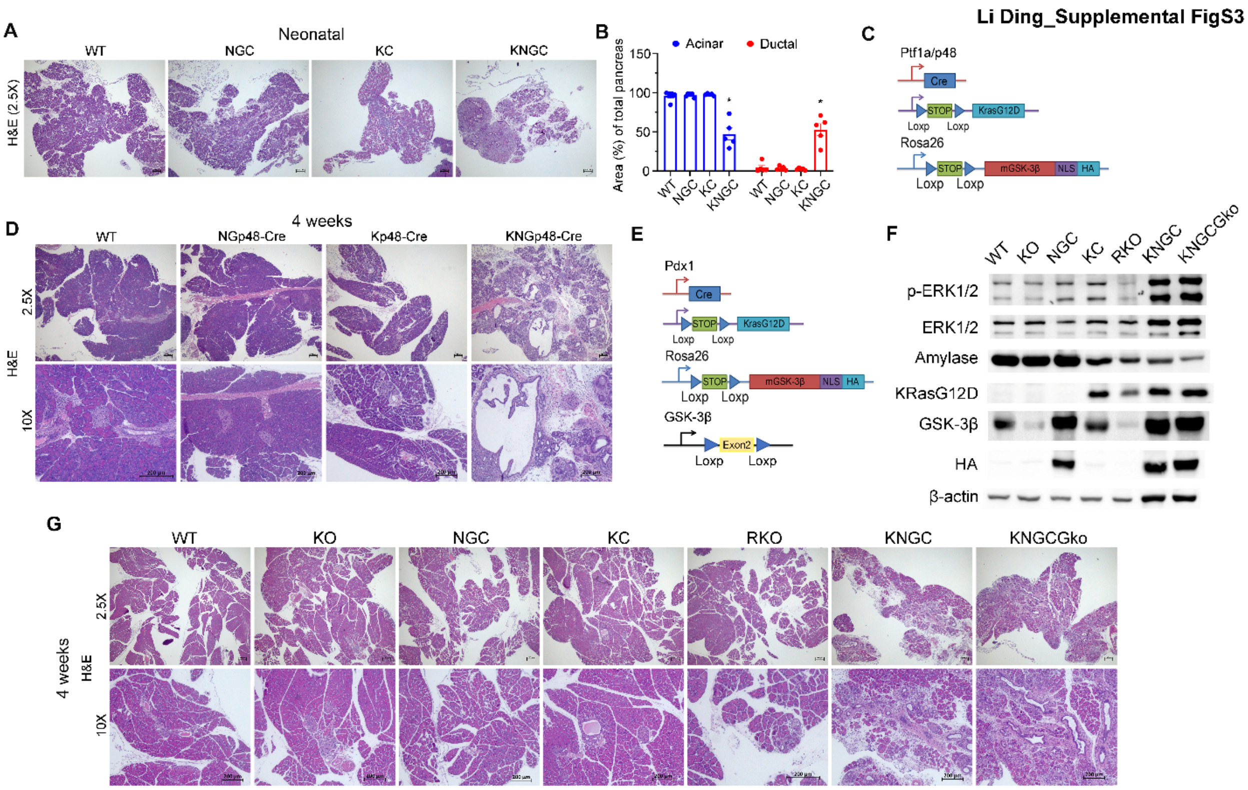

Figure S3. (A) H&E-stained pancreatic sections from indicated genotypes at age of neonatal. (B) Quantification of percentage of total pancreas area were analyzed and expressed as mean ± SEM. *P<0.05 KNGC mice versus the other genotypes. (C) Schematic representation of KNGp48-Cre (LSL-KRas^{G12D}/Rosa26-LSL-nuclear GSK-3β/p48-Cre) mouse model. Blue triangles indicate loxP sites. (D) H&E-stained pancreatic sections from KC and RKO mice. Black boxes indicate magnified area. (D) H&E-stained pancreatic sections from indicated genotypes at 4-week-old age. (E) Schematic representation of KNGCGko (LSL-KRas^{G12D}/Rosa26-LSL-nuclear GSK-3β/p48-Cre/GSK-3β$^{F/F}$) mouse model. Blue triangles indicate loxP sites. (F) Cell lysates from the indicated genotypes at 4-week-old were prepared and probed with the indicated antibodies. (G) H&E-stained pancreatic sections from indicated genotypes at 4-week-old age.

Li Ding_Supplemental FigS4

A

	NGC vs WT	KC vs WT	KNGC vs WT	KNGC vs NGC	KNGC vs KC
mRNAs increased	118	127	5304	4474	5056
mRNAs decreased	48	73	4593	4603	4297
Total	166	200	9897	9077	9353

B

	KEGG pathways with FDR<0.05	
	KNGC vs	
Cutoffs	NGC	KC
$Log_2FC > 2$	1131	1491
$Log_2FC > 3$	407	327
$Log_2FC < -2$	122	191
$Log_2FC < -3$	68	107

C **D**

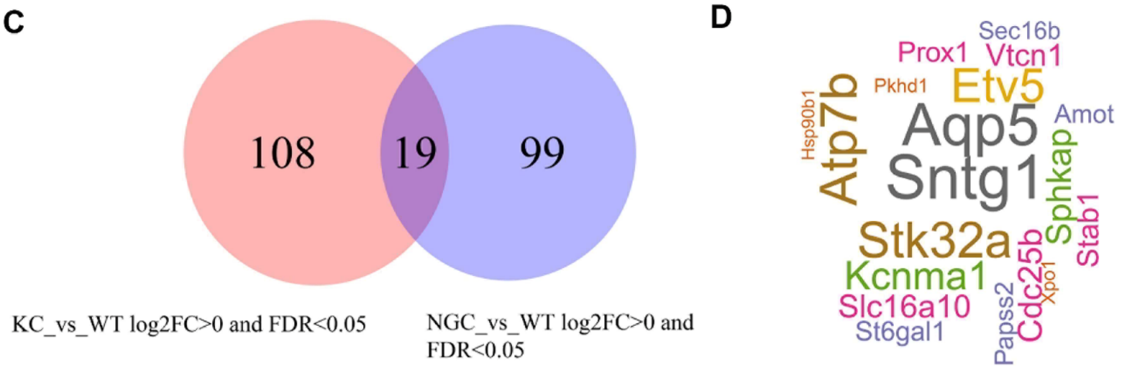

Figure S4. (A) Differential expression analyses by DESeq2 of RNA-Seq quantification of mRNA changes from different comparison as indicated, with an FDR cutoff of <0.05. (B) Number of enriched KEGG signaling pathways with FDR<0.05 in comparison between KNGC and NGC or KC mice, different log2 fold change (Log2FC) were used as cutoffs. (C) Venn diagram showing the number of genes fitting the indicated cutoffs. (D) Word cloud using 19 common genes shown in (C).

Li Ding_Supplemental FigS5

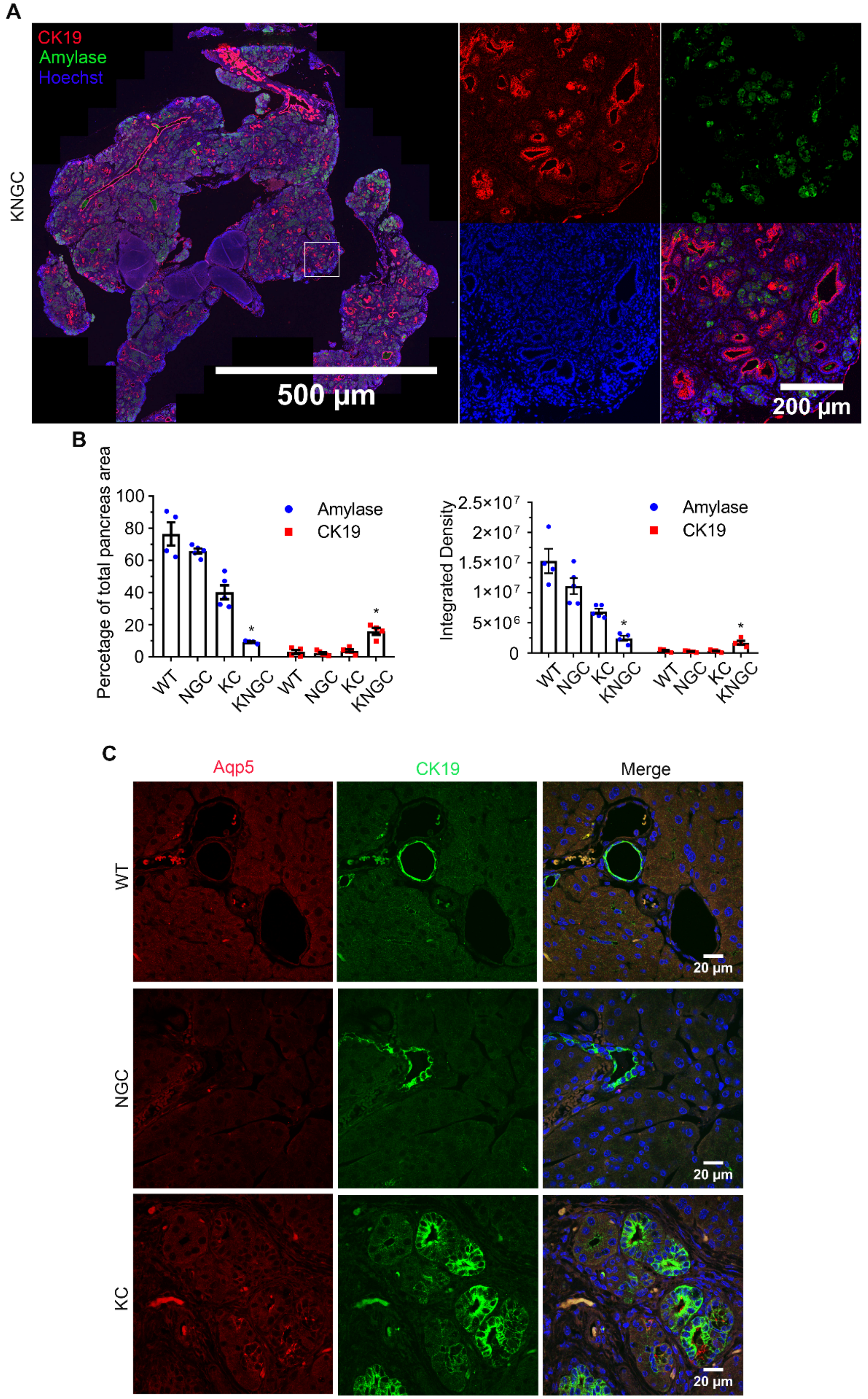

Figure S5. Transcriptional regulation of pancreatic ductal neoplasia by nuclear GSK-3β and $Kras^{G12D}$. (A) Immunofluorescence staining of CK19 (red) and Amylase (green) from pancreatic sections of 4-week-old KNGC mice. White boxes indicate magnified area. (B) Quantification of percentage of total pancreas area and integrated density were analyzed and expressed as mean ± SEM. *$P<0.05$ KNGC mice versus the other genotypes. (C) Immunofluorescence staining of Aqp5 (red) and CK19 (green) from pancreatic sections of 4-week-old WT, NGC and KC mice.

Li Ding_Supplemental FigS6

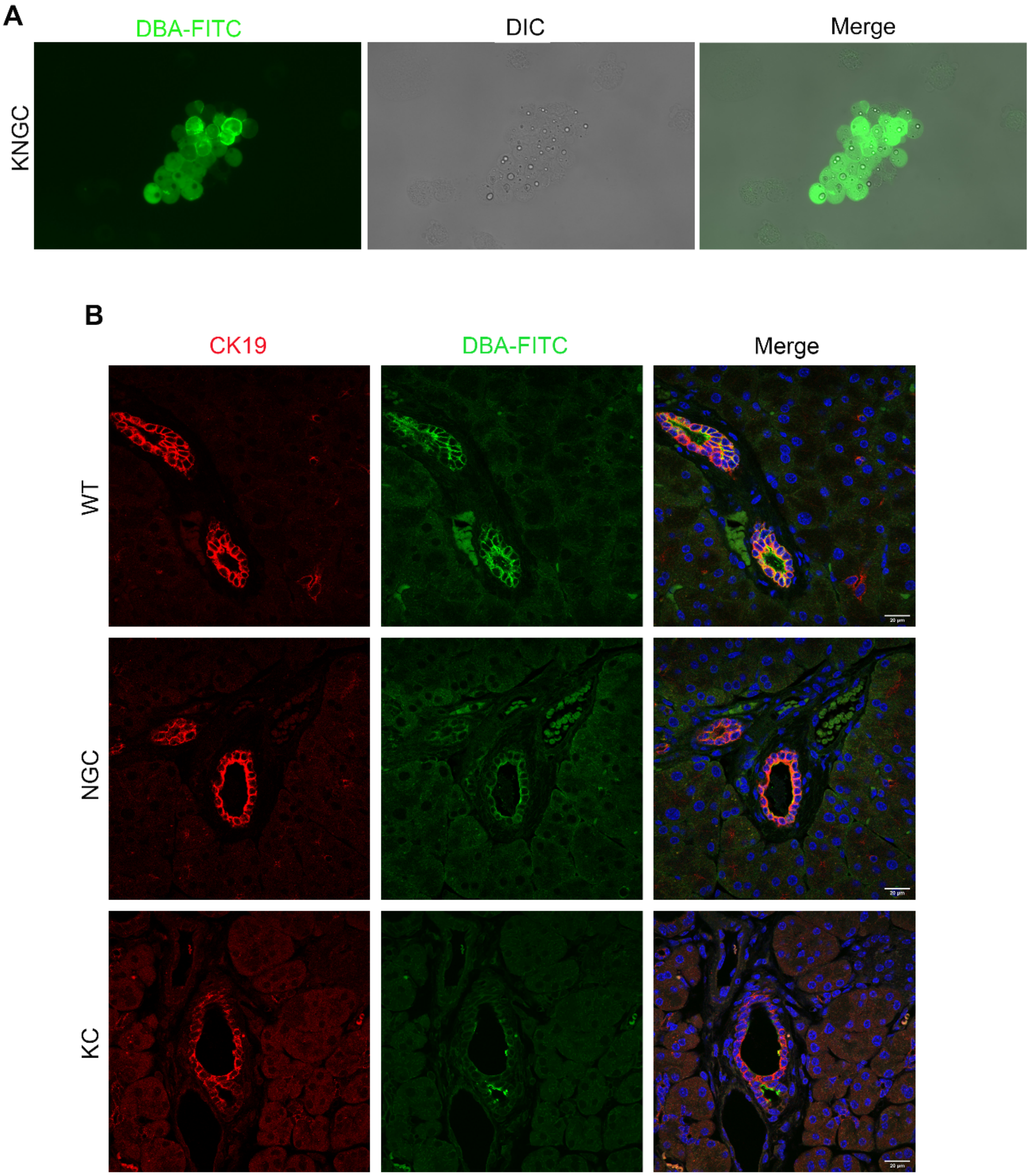

Figure S6. (A) Green fluorescent channel and differential interference contrast (DIC) image of isolated pancreatic cells from KNGC mice after negative selection, DBA-FITC labeling and anti-FITC-conjugated microbead separation. (B) Immunofluorescence staining of DBA-FITC (green) and CK19 (red) from pancreatic sections of 4-week-old WT, NGC and KC mice.

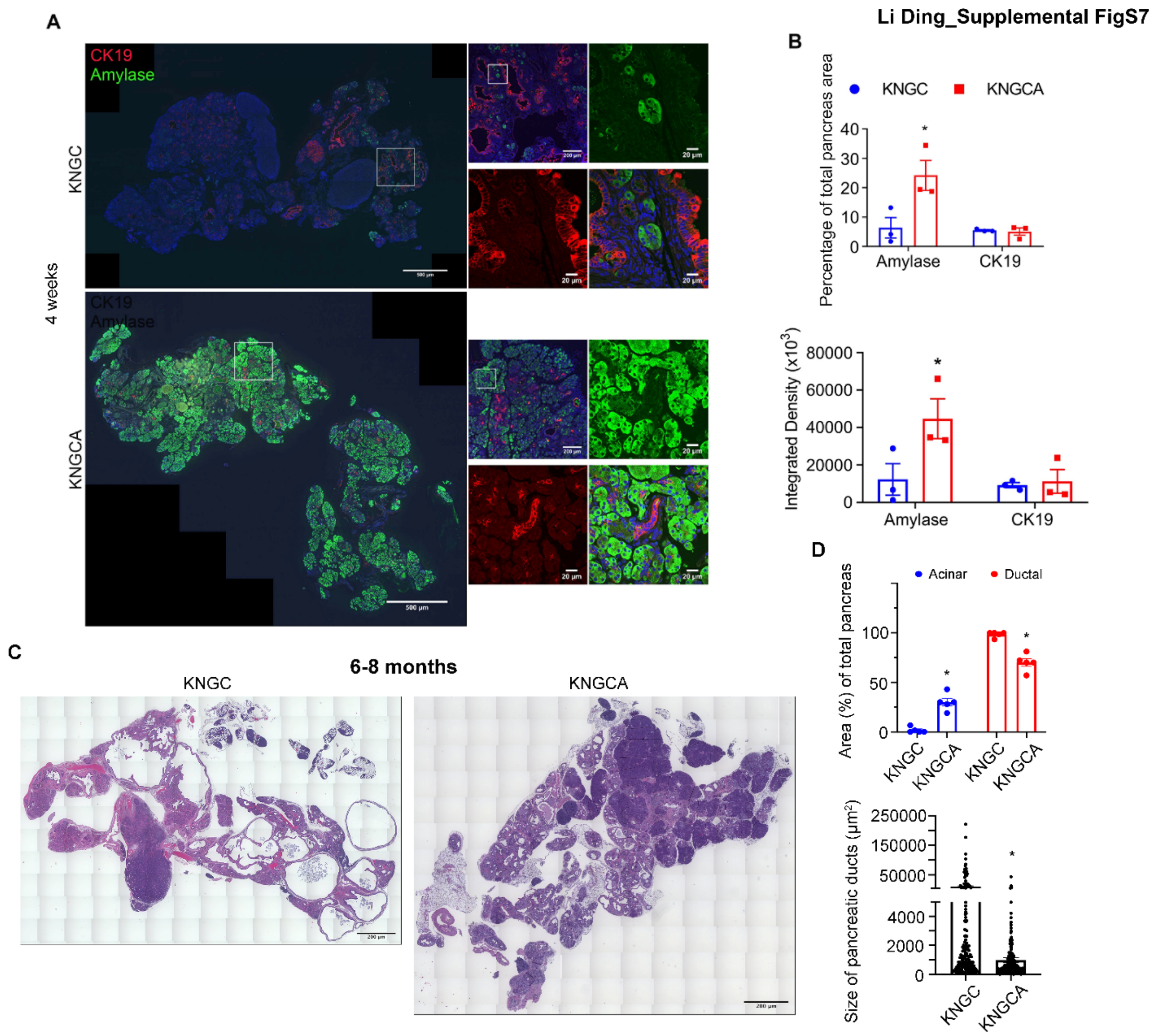

Figure S7. (A) Immunofluorescence staining of amylase (green) and CK19 (red) from pancreatic sections of KNGC and KNGCA mice at 4 weeks of age. White boxes indicate magnified area. Nuclei were counter-stained with Hoechst (blue). Shown are representative images. (B) Quantification of percentage and integrated density of Amylase and CK19 staining were analyzed and expressed as mean ± SEM. *P<0.05 KNGCA mice versus KNGC mice. (C) H&E-stained pancreatic sections from KNGC and KNGCA at the 6-8-month-old age. (B) Quantification of percentage of total pancreas

area and size of pancreatic ducts were analyzed and expressed as mean ± SEM. *P<0.05 KNGCA mice versus KNGC mice.

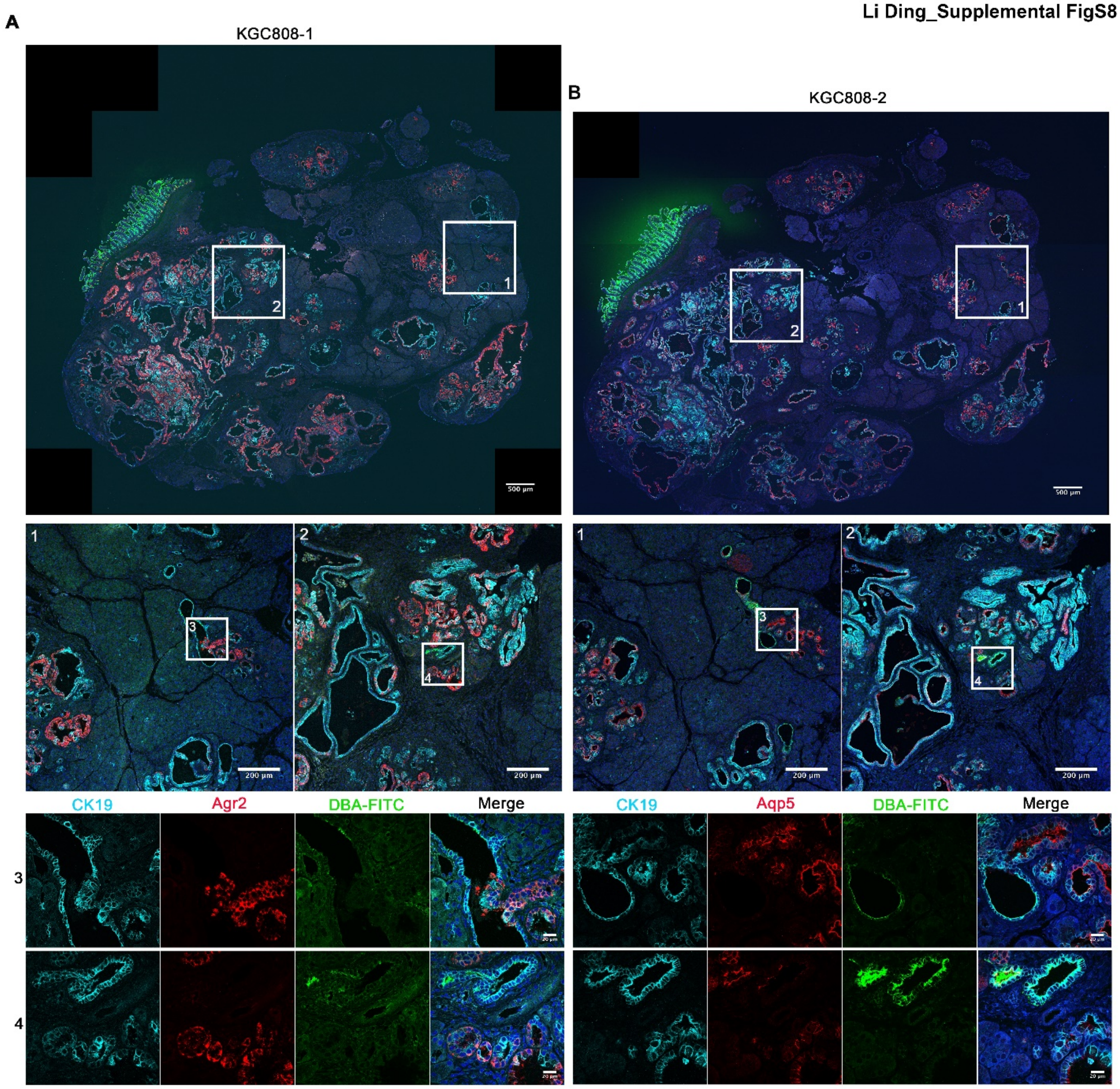

Figure S8. (A) Immunofluorescence staining of (A) Agr2 (red) and (B) Aqp5 (red) with DBA-FITC (green) and CK19 (cyan) from pancreatic serial sections of KGC mice at 11 weeks post Dox induction. White boxes indicate magnified area. Nuclei were counter-stained with Hoechst (blue).

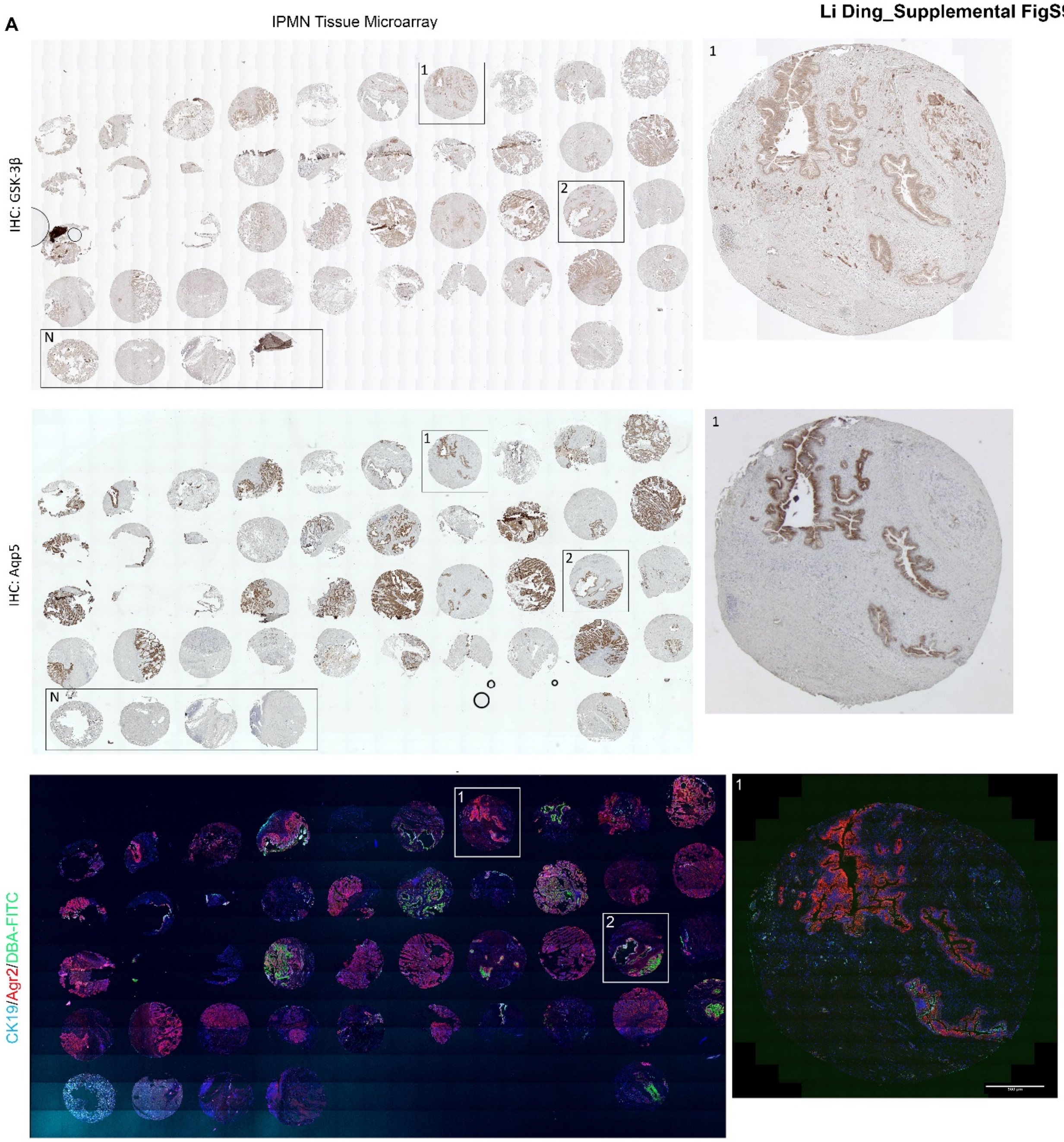

Figure S9. Immunohistochemistry staining of GSK-3β (Top), Aqp5 (Middle), and immunofluorescence staining of Agr2 (red) with DBA-FITC (green) and CK19 (cyan) (Bottom) from serial sections of a TMA containing human IPMN. White boxes indicated area magnified. Nuclei were counter-stained with Hoechst (blue).
